# High-resolution analysis of selection sweeps identified between fine-wool Merino and coarse-wool Churra sheep breeds

**DOI:** 10.1186/s12711-017-0354-x

**Published:** 2017-11-07

**Authors:** Beatriz Gutiérrez-Gil, Cristina Esteban-Blanco, Pamela Wiener, Praveen Krishna Chitneedi, Aroa Suarez-Vega, Juan-Jose Arranz

**Affiliations:** 1 Departamento de Producción Animal, Facultad de Veterinaria, Universidad de León, Campus de Vegazana s/n, León, 24071 Spain; 2 Fundación Centro Supercomputación de Castilla y León, Campus de Vegazana, León, 24071 Spain; 3 Roslin Institute and R(D)SVS, University of Edinburgh, Easter Bush Campus, Midlothian, EH25 9RG UK

## Abstract

**Background:**

With the aim of identifying selection signals in three Merino sheep lines that are highly specialized for fine wool production (Australian Industry Merino, Australian Merino and Australian Poll Merino) and considering that these lines have been subjected to selection not only for wool traits but also for growth and carcass traits and parasite resistance, we contrasted the OvineSNP50 BeadChip (50 K-chip) pooled genotypes of these Merino lines with the genotypes of a coarse-wool breed, phylogenetically related breed, Spanish Churra dairy sheep. Genome re-sequencing datasets of the two breeds were analyzed to further explore the genetic variation of the regions initially identified as putative selection signals.

**Results:**

Based on the 50 K-chip genotypes, we used the overlapping selection signals (SS) identified by four selection sweep mapping analyses (that detect genetic differentiation, reduced heterozygosity and patterns of haplotype diversity) to define 18 convergence candidate regions (CCR), five associated with positive selection in Australian Merino and the remainder indicating positive selection in Churra. Subsequent analysis of whole-genome sequences from 15 Churra and 13 Merino samples identified 142,400 genetic variants (139,745 bi-allelic SNPs and 2655 indels) within the 18 defined CCR. Annotation of 1291 variants that were significantly associated with breed identity between Churra and Merino samples identified 257 intragenic variants that caused 296 functional annotation variants, 275 of which were located across 31 coding genes. Among these, four synonymous and four missense variants (*NPR2_His847Arg*, *NCAPG_Ser585Phe*, *LCORL_Asp1214Glu* and *LCORL_Ile1441Leu*) were included.

**Conclusions:**

Here, we report the mapping and genetic variation of 18 selection signatures that were identified between Australian Merino and Spanish Churra sheep breeds, which were validated by an additional contrast between Spanish Merino and Churra genotypes. Analysis of whole-genome sequencing datasets allowed us to identify divergent variants that may be viewed as candidates involved in the phenotypic differences for wool, growth and meat production/quality traits between the breeds analyzed. The four missense variants located in the *NPR2*, *NCAPG* and *LCORL* genes may be related to selection sweep regions previously identified and various QTL reported in sheep in relation to growth traits and carcass composition.

**Electronic supplementary material:**

The online version of this article (doi:10.1186/s12711-017-0354-x) contains supplementary material, which is available to authorized users.

## Background

Approximately 5000 years ago, humans began to select sheep for desired characteristics (e.g., coat color, horns, meat, wool) which resulted in the development of different breeds [1]. Initially, sheep were reared mainly for meat; later, specialization for ‘secondary’ products, such as wool, emerged [2–4]. Sheep that have been selected for secondary products appear to have replaced the more primitive domestic populations. Selection for such phenotypes has left detectable signatures of selection within the genome of modern sheep. Due to the very strong selection intensity involved in animal breeding, these changes are expected to occur faster than those due to natural selection. Selection not only affects a favored mutation by rapidly increasing its frequency in the population, but it also produces a hitch-hiking effect of the frequency of neutral alleles at linked loci [5, 6]; these patterns in allele frequencies are known as selection signatures.

The signatures of selection in the genome, also known as selection sweeps, can be detected under the assumption that selection is locus-specific whereas other evolutionary forces such as random genetic drift, mutation and inbreeding, should be expressed genome-wide [7]. Hence, a variety of methods and statistics have been developed with the aim of identifying the selected loci at which allele frequencies have changed following a pattern that is consistent with positive selection. They can be based on between-population differentiation, reductions in local variability, deviations in the site frequency spectrum (SFS), and increases in linkage disequilibrium (LD) and extended haplotype structure [8–10]. Methods for detecting signatures of selection have historically been challenged by the confounding effects of demography; for example, recent population growth will result in an excess of rare variants compared to equilibrium expectation [11], and also recent and weak bottlenecks tend to mimic the effects of a selection sweep in several ways [12]. However, demographic events apply to the whole genome, whereas selective events affect different regions of the genome to various extents thanks to recombination [13]. This gives the possibility of distinguishing the two hypotheses by sampling several loci: a more or less common pattern is expected in the case of a bottleneck, while selective sweeps generate heterogeneity across loci [12]. Regions of low recombination may also produce an upward bias in the detection of signatures of selection, although apart from the bias issue, it should be noted that genuine selection sweeps in these regions will leave much stronger signals in regions of average recombination rate [14].

Another issue relates to the limitations of some selection mapping approaches; the standard approaches to detect signatures of selection consider “hard sweeps” where the new advantageous mutations spread rapidly to fixation, purging variation at linked sites as they spread. However, recent studies highlight the potential importance of ‘soft sweeps’, i.e., sweeps from standing variation, or sweeps in which multiple mutations start to sweep simultaneously at a single locus [15]. Soft sweeps, which are often related to adaptation, leave more subtle signatures in the genome (e.g. diversity is not necessarily reduced in the vicinity of the adaptive locus as with hard sweeps) and thus are more difficult to detect [16].

In past years, many genome screening studies based on high-density, genome-wide single nucleotide polymorphism (SNP) panels (i.e., SNP-chips) have been conducted with the goal of detecting signatures of selection in livestock species [17–19]. More recently, whole-genome re-sequencing has emerged as an economically feasible tool for assessing genomic variation within and among populations, and the large-scale information derived from the new sequencing technologies can be exploited to identify signatures of selection [20] or further explore previously detected signatures of selection.

The Sheep HapMap project, for which genotypes were generated from 3004 domestic sheep from 71 breeds using the Illumina OvineSNP50K BeadChip assay (50 K-chip), generated valuable information that can be used to perform analyses of signatures of selection in sheep [21]. Global analyses of genetic differentiation in the Sheep HapMap dataset identified several genomic regions that contained genes for coat pigmentation, skeletal morphology, body size, growth, and reproduction [21, 22]. Many of these regions were later confirmed by haplotype-based selection sweep mapping [23, 24]. Also based on this dataset, as well as additional information in some cases, signatures of selection have been reported in thin and fat tail sheep breeds [25] and in specialized European dairy sheep breeds [19]. Further selection mapping studies have identified signatures of selection related to resistance/susceptibility to gastrointestinal nematodes [26], adaptation to different ecoregions [27] or climate adaptation [28, 29]. Information from additional studies using the 50 K-chip to study the biodiversity of sheep breeds [30–32] can also help to extend our current knowledge on the ovine genomic regions that have been affected by human-driven selection.

In sheep, selection for wool traits has been extensively carried out for several centuries. Spanish Merino, which was developed since the late Middle Ages [33], appears to have originated during Roman times through the introduction of fine wool ewes from the Southern Italian region of Apulia into Spain and the later selection for white wool color through crosses with African rams imported by Arabs [34] at the beginning of the Middle Ages. Due to the value of their fiber, the Honourable Council of the Mesta strongly protected Merino flocks, and their exportation was strictly forbidden for several centuries. Removal of these restrictions in the eighteenth century led to the dispersal of Merino sheep to Eastern Europe, China, Australia and New Zealand [34]. The first Merino sheep were introduced from South Africa into Australia in 1797 [35]. In this country, intensive selective breeding has enhanced the already fine quality of the wool to produce Australian Merino wool, which based on its long, fine fibers, enables the production of lighter and softer wool fabrics. Hence by 1870, Australian Merino wool industry was the global leader in both the quantity and quality of its wool production. However, in addition to wool, Australian Merino also plays an important role in lamb meat production. Over the last two decades, the Australian sheep meat industry has delivered large increases in lamb production and profitability, with genetic improvement in growth, leanness and muscling contributing substantially to these gains [36, 37]. Australian Merino flocks have also been selected for disease resistance, in particular, by focusing on gastrointestinal nematode parasites, flystrike (cutaneous myiasis) and footrot [38]. Specifically, the three Merino lines considered in this paper have been subjected to selection pressure to reduce susceptibility to parasites [21].

The goal of our work was the identification of regions of selection sweeps related to traits for which Australian fine-wool Merino breeds have been selected. Considering the Iberian origin of Merino breeds and the estimated divergence time between sheep breeds derived from the analysis of the Sheep HapMap project dataset [21], we analyzed genome-wide SNP information from three Australian Merino breeds that are highly specialized for the production of fine wool (Australian Industry Merino, Australian Merino and Australian Poll Merino) and the related coarse-wool breed, Spanish Churra. This is an autochthonous double-purpose breed of the northwest region of Castilla y León in Spain. Traditional Churra flocks are managed based on an intermediate level of dairy specialization (the dairy breeding program was started in 1986 [39]) with a variable fraction of the farm income derived from the sale of suckling lamb meat. The milk is used to produce cheese of high quality value, which is covered by a protected geographical indication (PGI) [40].

With the aim of further exploring the genetic variation in genomic regions that show evidence of selection, whole-genome sequence data from Churra and Australian Merino samples were subsequently analyzed. By identifying the SNPs within the regions of interest that exhibited the most extreme divergence in allele frequencies between the Churra and Merino datasets, this study provides a detailed survey of the genetic variation that underlies the identified regions of selection sweeps.

## Methods

### Mapping of selection sweeps

#### Breed selection

**Fig. 1:**
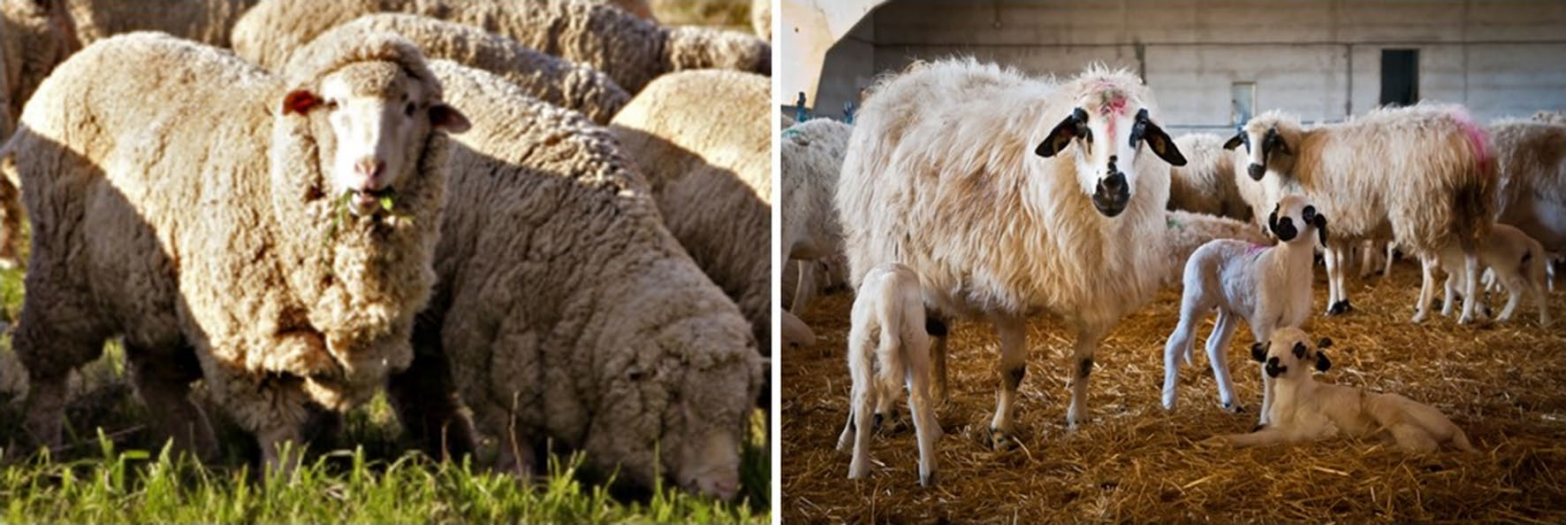
Sheep breeds selected for this study, Australian Merino (left) and Spanish Churra (right). Original images taken from Wikipedia (https://commons.wikimedia.org/w/index.php?curid=12599612; https://commons.wikimedia.org/w/index.php?curid=12174588)

In order to identify selection sweeps related to fine-wool production, three Merino lines described as “with extreme fine wool” from the International Sheep Genomics (ISGC) dataset were included in this study. According to the divergence times estimated for the breeds included within the Sheep HapMap project, based on LD and haplotype sharing, these three Merino lines show a recent divergence time (0 to 80 generations) (Figure S10 and Fig. 3 from Kijas et al. [21]). With the aim of providing an appropriate comparison to identify signatures of selection related to wool production, we selected the Spanish Churra sheep breed, which is a coarse-wool breed related to Merino, as shown by the population analysis reported by Fariello et al. [22] in which these two breeds are grouped together within the defined South West European group. According to a haplotype-sharing analysis, Churra sheep show a short and consistent divergence time with each of the three Merino lines (160 to 240 years, see Figure S10 and Fig. 3 from Ref [21]), which supports our study design in which the three Australian sheep breeds selected are considered together against Churra sheep. In addition to wool characteristics, Merino and Churra sheep differ in other traits (see Additional file 1: Table S1) (Fig. 1). Briefly, adult animals of the Australian Merino lines are larger than Churra sheep individuals, whereas weight at birth is similar in the two breeds. Both breeds show white wool color although Churra sheep show characteristic black patches around the eyes, ears and the ends of legs. Note that because the two breeds differ in various traits, the signatures of selection identified here may be related not only to wool traits but also to other phenotypes for which the selection pressure performed in the two breeds differs. Considering the possibility that the geographical isolation and distance between the two studied populations could be a confounding effect for the identified selection sweeps, we performed additional validation analyses by contrasting Churra and Spanish Merino breeds.

#### Genotypes, quality control and analysis of population structure

We included in this work an initial subset of SNP genotypes for the ovine 50 K-chip that were generated within the framework of the Sheep HapMap project [21], and which are available upon request (http://www.sheephapmap.org/termsofaccess.php). The extracted subset included 332 samples from the Australian Industry Merino (n = 88), Australian Merino (n = 50), Australian Poll Merino (n = 98) and Spanish Churra (n = 96) breeds. In addition, 184 DNA samples of Churra sires included in the selection nucleus of the National Association of Spanish Churra Breeders were also genotyped with the same SNP array. These samples were extracted from semen samples following a classical phenol–chloroform DNA extraction protocol [41], and genotyped by an external laboratory service. The raw genotypes for the 54,241 SNPs included in the genotyping platform were first analyzed with the GenomeStudio software (Illumina) (GenCall score for raw genotypes > 0.15) which was used to extract the genotypes in standard format for the Plink_v1.09 software [42].

The HapMap project samples had already been subjected to quality control (QC) filtering [21], resulting in 49,034 SNPs available for analysis. To join the two separate datasets, we first merged the new Churra dataset (n = 184; 54,241 SNPs) and the HapMap dataset (n = 332; 49,304 SNPs) based on the common SNPs. We then selected the SNPs that mapped, with positions based on sheep genome assembly Oar_v3.1 [43], on the ovine autosomes, resulting in 47,415 SNPs. This dataset was then subjected to the following filtering criteria: (1) individual call rate higher than 90% (two Churra individuals genotyped by our group were removed) and (2) marker call rate higher than 90% (28 SNPs removed due to missing genotype data). Hence, 514 individuals (Churra = 278 and Merino = 236) and 47,387 SNPs were available for further analyses.

To evaluate the genetic structure of the data and confirm the number of different genetic populations, the genotypes of the three Merino fine wool breeds and the genotypes of the Churra individuals were analysed by principal component analysis (PCA) of allele sharing (using smartpca, implemented in Eigensoft [44]), and ancestry estimation (Admixture software [45]). The results of these analyses identified two clearly distinct genetic populations, corresponding to the Merino group and Churra sheep, for details on these analyses and description of results (see Additional file 2 and Additional file 3: Figures S1, S2 and S3). Genotypes were pooled into a single Australian Merino dataset for the three Australian Merino populations.

In addition, as a further validation analysis, we compared the genotypes of 20 randomly chosen Churra samples from the Sheep HapMap dataset and the 20 Spanish Merino samples genotyped by Ciani et al. [34].

#### Identification of candidate regions under selection using individual analyses

Several analyses between the complete set of Spanish Churra and Australian Merino genotypes were performed to detect candidate regions that harbor signatures of selection. First, a genetic differentiation analysis was used to contrast the Australian Merino and Churra genotypes by calculating the unbiased estimate of Weir and Cockerham’s *F*_ST_ [8] for each SNP, as described by Akey et al. [46]. In a second analysis, regions of reduced heterozygosity in the two groups were identified by estimating the observed heterozygosity (ObsHtz) for each SNP. For these two analyses, *F*_ST_ and ObsHtz values estimated for each SNP were each averaged across a sliding window of nine SNPs (e.g., *F*_ST__9SNPW). The size of the sliding window was based on a previous analysis by Gutierrez-Gil et al. [19] for a test control region encompassing the *myostatin* (*GDF*-*8*) gene, which is known to have been under selection in the Texel breed. The identification of candidate signatures of selection in each of the individual analyses was based on window estimates at the extreme of the empirical distributions, as suggested by Akey et al. [46] and has been used in a number of subsequent studies [18, 19, 47–49]. Specifically, we considered that a position carried a signature of selection if it was in the top 0.5th percent of the distributions for genetic differentiation (*F*_ST_) or the bottom 0.5th percent for observed heterozygosity. The distribution of the physical sizes of windows based on the 9-SNP fixed-size criteria (average window size = 411.71 kb; average distance between central SNPs of consecutive windows = 51.53 kb) was found to be fairly narrow (98.28% of the windows were 200 to 600 kb long; only 0.74% of the windows were longer than 1000 kb) and thus should provide reasonable estimates of local genomic diversity, in contrast to analyses based on a low-density chip [50].

As a complementary approach to map selection sweeps, we used the hapflk_v1.3 software (https://forge-dga.jouy.inra.fr/projects/hapflk), which implements the FLK [51] and hapFLK [23] tests. The FLK metric tests the neutrality of polymorphic markers by contrasting their allele frequencies in a set of populations against what is expected under a neutral evolution scenario. The hapFLK statistic extends the FLK test to account for the differences in haplotype frequencies between populations. This method has been shown to be robust with respect to bottlenecks and migration [23]. To run the hapflk analysis, the Reynolds’ distances between the Churra and Merino populations were converted to a kinship matrix with an R script provided by the hapFLK developers (available at https://forge-dga.jouy.inra.fr/projects/hapflk/documents). Subsequently, by assuming 20 haplotype clusters in the LD model (-K 20; number of haplotype clusters determined by running a fastPHASE cross-validation analysis), the hapFLK statistics were later computed and averaged across 30 EM runs to fit the LD model (–nfit = 30). The standardization of the statistics using the corresponding python script provided with the software allowed the estimation of the associated *P* values from a standard normal distribution. To correct for multiple testing, we considered the threshold of the nominal *P* value as < 0.001 to identify the significant haplotypes, following previous studies using hapFLK analysis on the Sheep HapMap dataset [22, 24].

In addition, we used the *rehh* software [52] to perform an additional analysis based on the cross-population extended haplotype homozygosity (XP-EHH) test defined by Sabeti et al. [53]. This statistic compares the EHH profiles for bi-allelic SNPs between two populations and is defined, for a given allele, as the log of the ratio of the integrals of the EHH profiles between the two populations. The comparison between populations normalizes the effects of large-scale variation in recombination rates on haplotype diversity and has a high statistical power to detect sweeps that are close to fixation [53]. Alleles were designated at each locus as either minor (“1”, “ancestral”) or major (“2”, “derived”), based on their allele frequency in the overall population. Positive and negative XP-EHH estimates indicated positive recent selection in Churra and Merino, respectively. Based on the *P* values supplied by *rehh*, and for consistency with the threshold previously used for hapFLK, we considered as significant those positions showing a *P* value less than 0.001.

For the four selection sweep mapping analyses, positions that showed evidence of selection (i.e. included in the top/bottom 0.5th percent of the corresponding distribution or showing a *P* value less than 0.001) and within 0.150 Mb of each other were considered to be the result of the same selection sweep and were labeled, depending on the analysis method, as *F*_ST_-SS, Merino-ObsHtz-SS, Churra-ObsHtz-SS, hapfFLK-SS and XP-EHH-SS. This criterion to connect identified SNPs into discrete regions was established based on an exploratory analysis of the extent of LD and the haplotype block structure of the Churra and Merino populations. Based on the results of LD analysis performed with Haploview_v4.2 [54] (for details see Additional file 4 and Additional file 5: Figure S4), and following Tang et al. [55], we initially considered regions of 50 kb (based on the fact that among the identified haplotype blocks, the proportion of blocks of size 50 kb or more was 43.65% and 50.59% in Merino and Churra, respectively) and extended these regions by 50 kb in both directions [based on the fact that the estimation of half-length decay in LD in the two breeds was around 50 kb (see Additional file 4)].

The four selection sweep mapping analyses described above were subsequently performed on the 20 Spanish Churra and 20 Spanish Merino samples selected for the validation analysis, using the same criteria to identify positions showing evidence of selection and to group these positions into selection sweeps.

#### Identification of shared regions across methods

Considering the results obtained in the Australian Merino versus Churra analyses, those regions showing an overlap between at least one of the two methods based on haplotype analysis (hapFLK and XP-EHH) and at least one of the two other considered methods (*F*_ST_ and ObsHz), were labeled as convergence candidate regions (CCR). The coordinates of the identified CCR were compared with previously reported ovine selective sweeps and previously described sheep QTL in these regions based on the Animal QTLdatabase [56]. An initial assessment of possible functional candidate genes that mapped within the identified CCR was performed using Ensembl BioMart [57] to extract the annotated genes for the relevant genomic intervals. The list of extracted genes was later contrasted with the list of 1255 genes provided by Gutierrez-Gil et al. [17], which are candidates for selection in cattle (and other livestock species) due to their known association with physical features (horns, stature, body size and coat color) or production traits (milk production, mastitis, and meat production/quality traits). This list was extended to include 148 candidate genes for wool production/quality, such as those related to hair follicle cycling (reviewed by Stenn and Paus [58]), or identified as associated with wool production/quality by genome-wide association studies (GWAS) [59] or differential expression analysis [60] (see Additional file 6: Table S2).

The same overlapping criteria were applied to the results from the validation analyses and the resulting convergence candidate regions were labelled as CCR_(Churra20-SpanishMerino20)_.

### High-resolution analysis of selection sweep regions

#### Whole-genome sequencing (WGSeq) data

WGSeq data for 13 Australian Merino and 15 Churra samples were analysed in this study. Below, we summarize the detailed description and source of the analyzed datasets, which are in Additional file 7: Table S3. Briefly, 13 of the Churra samples were sequenced by our research group and ANCHE (National Association of Breeders of Spanish Churra sheep). These samples included males from the selection nucleus of ANCHE with the largest number of daughters in the general commercial population of Spanish Churra dairy sheep. For these samples, the bam files of the reads mapping to the 18 CCR identified in this work are available in the sequence read archive (SRA) repository [24] within the Bioproject PRJNA395499. In addition, we included in our study publicly available WGSeq datasets from two different projects of the SRA repository: (a) sequencing data for two Churra and three Australian Merino samples were obtained from the “*Ovis aries* diversity study” (PRJNA160933), coordinated by the International Sheep Genomics Consortium as an extension of the Sheep HapMap project; and (b) WGSeq data for 10 Australian Merino samples generated within the “Australian CRC for Sheep Industry Innovation whole-genome sequence collection” project (PRJNA325682) carried out by the Sheep Commonwealth Government’s Cooperative Research Centres (SheepCRC). All sequencing data were generated with paired-end Illumina technology (Illumina HiSeq 2000 and Hiseq 2500 sequencers).

#### Bioinformatics analysis

For the samples obtained from the SRA repository, the SRA-Toolkit [61] was used to convert the data to FASTQ format. Then, a common workflow was performed for all 28 WGSeq datasets. Following the criteria of Kijas et al. [62] for the identification of high-quality allelic variants within Run 1 of the Sheep genomes project (PRJEB14685 at the European Variant Archive, EVA), we performed the following five steps to identify allelic variants using GATK [63] and Samtools [64] software: (1) quality of the raw reads was assessed with the FastQC program [65]; (2) the low-quality reads were filtered with Trimmomatic [66] using filter options for paired end samples (-phred33, LEADING:5, TRAILING:5 SLIDINGWINDOW:4:20, MINLEN:36 ILLUMINACLIP: Trimmomatic-0.33/adapters/TruSeq 3-PE.fa:2:30:10); (3) alignment of samples against the reference genome OAR_v3.1 [43] with the Burrows-Wheeler aligner (BWA) [67] using the maximal exact matches (*mem*) mapping function; (4) data manipulation and preliminary statistical analysis using SAMtools [64, 68] (i.e. transformation of*.sam* files into*.bam* binary format and removal of non-mapped reads and the estimation of alignment statistics), the Picard program [69] (i.e. sorting reads, removal of duplicate reads and index building) and Genome Analysis ToolKit v3.3.0 (GATK) [63] (base quality score re-calibration and indel re-alignment); and (v) considering the reads that mapped to the 18 genomic intervals defined as CCR, a variant calling analysis of the 28 samples was done using two different algorithms: the Samtools mpileup [64, 68] analysis, using the default detection parameters, and the GATK HaplotypeCaller tool [63], using default parameters, as suggested in GATK Best Practices recommendations [70]. Using the snpSIFT software [71], filters were applied independently to each of the Samtools and GATK produced VCF files to remove lower quality variants (DP > 10, QUAL > 30, MQ > 30, QD > 5 and FS < 60). An intersect set for the 28 samples, containing those variants concordant between Samtools and GATK predictions, was extracted using BCFtools [68, 72] to produce the final VCF file.

#### Identification and study of divergent variability in the candidate regions

Among the variants localized in the targeted regions, we selected the SNPs that showed the most significant association with the breed identity between the Churra and Australian Merino samples. To select these SNPs, we first used the VCF-tools software [73] to filter only the SNPs that were detected in all variants and to convert the dataset into PLINK format [42]. Using the PLINK software, we first performed a quality control step on the raw genotypes by discarding SNPs and individuals with genotyping call rates lower than 90%, and SNPs with a minor allele frequency (MAF) lower than 0.01 (--*mind 0.1* --*geno 0.1* --maf 0.01). We then performed a Chi square association test (using the --*assoc* option) to identify the SNPs that showed the most significant associations with breed identity and therefore that had the most extreme divergent allele frequencies between the compared populations (e.g. SNPs with genotype “11” in Churra and “22” in Merino, or vice versa). For those SNPs with significant Bonferroni-corrected *P* values, (considering the number of independent tests as the total number of tested SNPs considered), we performed a functional annotation analysis to assess the possible biological impact of the considered mutations using the Ensembl Variant Effect Predictor (VEP) software [74] (based on the annotated genes of the Oar_v3.1 reference genome). For the non-synonymous variants, the results of the SIFT software analysis [75] regarding predicted effect on protein function were obtained from Ensembl. When the functional analysis assigned one of the divergent variants to a novel gene or pseudogene, we performed BLASTN searches (based on the ± 1.500 bp interval, centered at the SNP location) to identify orthologous genes in cattle (*Bos taurus*) and/or human (*Homo sapiens*) genomes. For one of the novel genes that harbored missense mutations, a BLASTN analysis was performed against the newly updated sheep reference genome (Oar_4.0) [76].

## Results

### Identification of candidate selection sweeps between Australian Merino and Churra sheep based on individual methods

#### Candidate regions identified by the genetic differentiation analysis

**Fig. 2:**
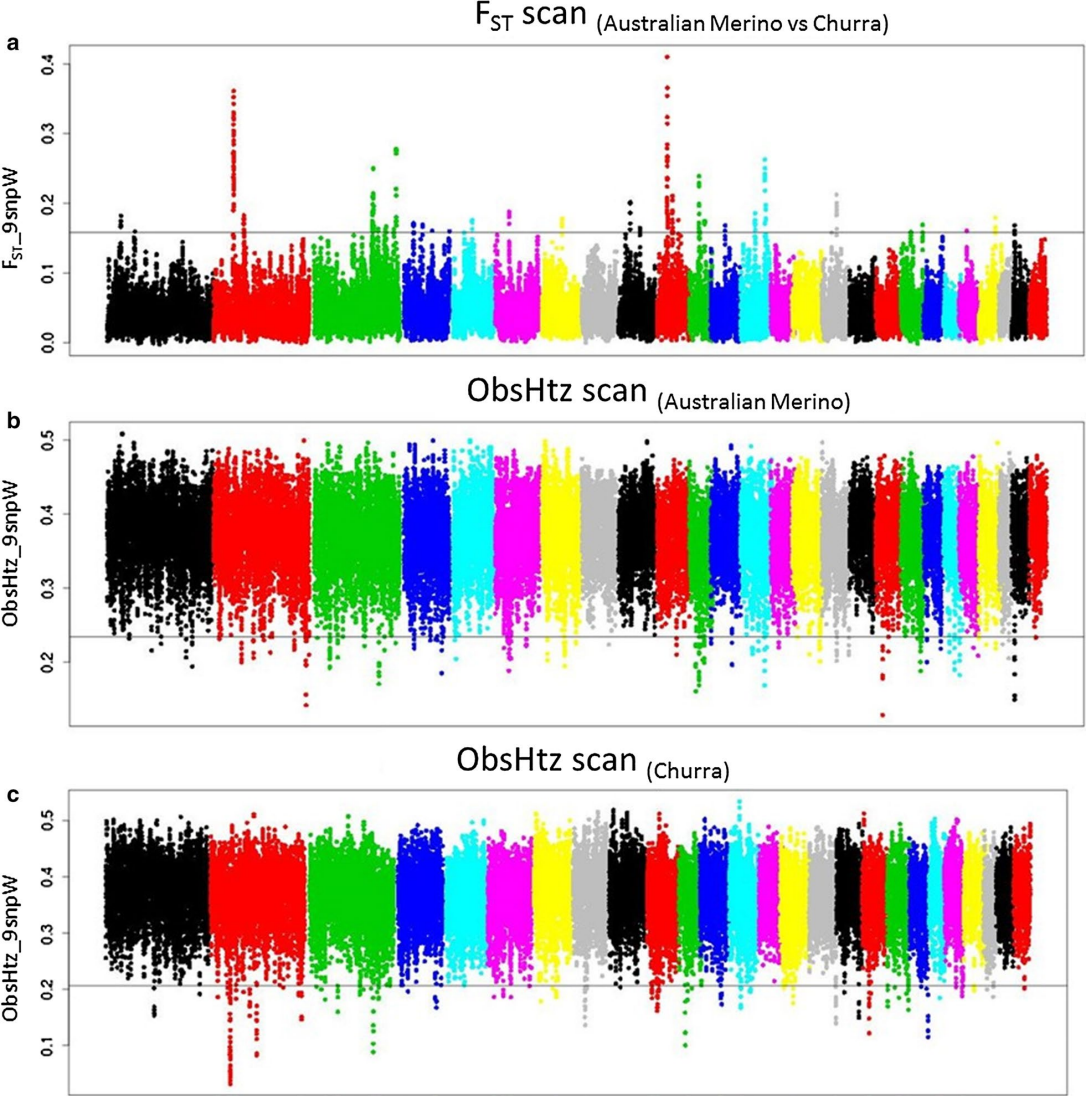
Results of the genetic differentiation analysis (**a**) and of the analysis of reduced heterozygosity in the Australian Merino populations studied here (Australian Industry Merino, Australian Merino and Australian Poll Merino) (**b**) and Spanish Churra sheepAustralian Merino sheep (**c**). **a** *F*_ST_ values obtained across the whole-genome (averaged in sliding 9-SNP windows) when contrasting the 50 K-chip pooled genotypes of the Australian Merino and Spanish Churra sheep samples considered in this work. The horizontal line indicates the top 0.5th percent threshold of the *F*_ST_-distribution. **b**, **c** Genome-wide distribution of observed heterozygosity values (averaged over a sliding 9-SNP window) for the pooled genotypes of the three Australian Merino populations (**b**) and Spanish Churra (**c**). The horizontal lines indicate the bottom 0.5th percent thresholds of the heterozygosity distributions

Of the *F*_ST_ values averaged in sliding windows of nine SNPs (Fig. 2a), the top 0.5% included 236 values, ranging from 0.119 to 0.325. Following the criteria previously described i.e. allowing a maximum gap of 0.150 Mb to define an *F*_ST_-SS, 49 genomic regions, distributed over 17 autosomes, were considered as potential selection sweeps (*F*_ST_-SS1 to *F*_ST_-SS49; Fig. 2a and see Additional file 8: Table S4). The largest number of *F*_ST_-SS was on chromosome 3 (OAR3, OAR for *Ovis aries*), where 12 signals were labelled as signatures of selection. The length of the labelled *F*_ST_-SS regions varied from a single central tested SNP (including the averaged estimates of the corresponding 9-SNP window), for 17 regions, to one region involving 40 windows, spanning 1.699 Mb and including 40 SNPs (OAR2, *F*_ST_-SS4).

#### Candidate regions identified based on reduced heterozygosity

Ninety-six Merino-ObsHtz-SS, distributed across 24 autosomes, were identified after grouping the bottom 0.5% values of the ObsHtz distribution (Fig. 2b; and see Additional file 9: Table S5). The largest candidate region identified by this analysis was located at the proximal end of OAR11 and spanned 1.120 Mb (Merino ObsHtz-SS58: 0.000012–1.120037 Mb). This region included information from 19 central tested SNPs while 45 of the Merino-ObsHtz-SS regions were defined by a single central tested SNP (including the averaged estimates of the corresponding 9-SNP window). When the same analysis was performed with the Churra genotypes, 72 genomic regions (over 23 autosomes) were identified based on the positions included in the bottom 0.5% of the ObsHtz distribution (Churra-ObsHtz-SS) (Fig. 2c; and see Additional file 10: Table S6). The largest of these regions, found on OAR2 (Churra-ObsHtz-SS5: 51.898–52.998 Mb), spanned 1.10 Mb and involved 28 9-SNP windows (i.e. 28 central tested SNPs) while 29 Churra-ObsHtz-SS were based on the averaged ObsHtz estimate assigned to single SNP position.

#### Candidate regions identified based on haplotype-based analyses

**Fig. 3:**
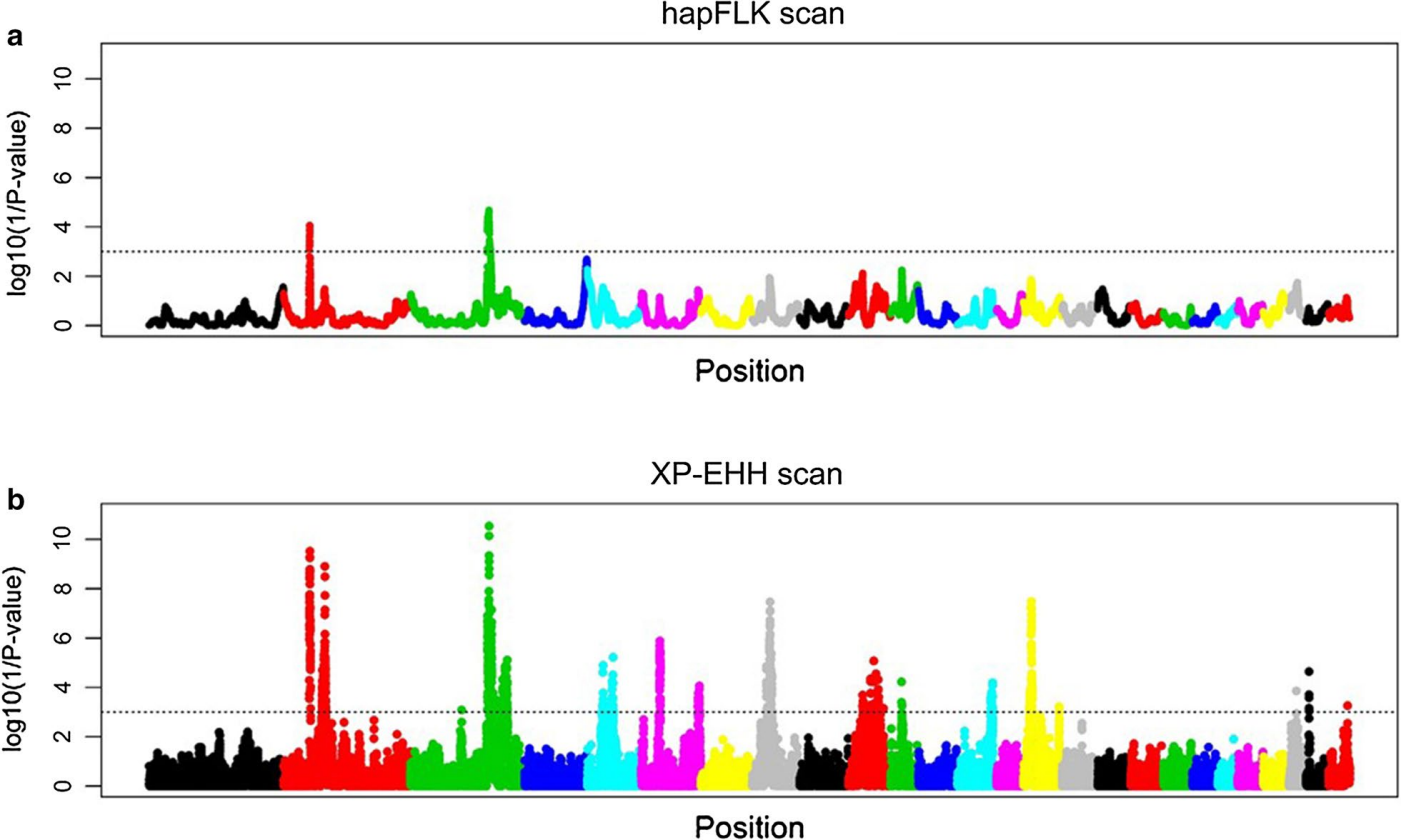
Results of the selection sweep mapping analyses performed with the two haplotype-based methods used in this work, performed with the hapFLK (**a**) and the *rehh* (XP-EHH analysis) (**b**) software. Genome-wide distribution of the log (1/*P* value) obtained from each analysis are represented on the Y-axis. The horizontal lines represent the significance threshold considered (*P* < 0.001)

The hapFLK analysis identified seven significant regions (*P* value < 0.001), one located on OAR2 (hapFLK-SS-1) and the rest located on OAR3 (Fig. 3a; and see Additional file 11: Table S7). The longest selection sweep identified by this approach was hapFLK-SS-6, located on OAR3 (153.963–155.382 Mb). Two other candidate regions involved also an interval longer than 1 Mb: hapFLK-SS-1 (OAR2: 51.898–52.939 Mb) and hapFLK-SS-3 (OAR3: 151.088–152.393 Mb). The XP-EHH analysis identified 98 significant selection sweeps (*P* value < 0.001) (distributed over 12 autosomes) (Fig. 3b; and see Additional file 12: Table S8). Only six of them, located on OAR6, 11, 15 and 25, showed signatures of selection in the Merino group, whereas the remainder were identified in Churra. Seven of the regions detected by this analysis covered an interval longer than 1 Mb, with the longest selection sweep (XP-EHH-SS17) located on OAR3 (154.638–158.340 Mb). Of the significant regions identified by this analysis, 25 involved a single SNP position.

#### Convergence of results from the different analyses

Eighteen genomic regions were labelled as convergence candidate regions (CCR) based on the overlap of significant results based on at least one method of each of the two types of analyses performed (i.e. based on allele/genotype frequencies and on haplotype-based information). These regions were located on OAR2, 3, 6, 8, 10, 11, 15 and 25 (Table 1). The intervals included in these CCR ranged from 18.113 kb (CCR17 on OAR15) to 3.701264 Mb (CCR6 on OAR3). All the labeled convergence regions involved a significant result from the XP-EHH analysis, with a good concordance between the sign of the XP-EHH score and the regions showing a reduction in heterozygosity in Merino or Churra. Hence, five of the labeled CCR were related to positive selection in Merino and the other 13 were related to positive selection in Churra.

**Table 1:** Convergence regions identified in this study based on the overlapping of the results of the four mapping analyses performed to identify selection sweeps between Churra and Australian Merino breeds

CCR^a^	SS^b^	Chr^c^	CCR flanking markers	Start position (bp)	End position (bp)	XP-EHH value^d^
1	*XPEHH*-*SS1*	*2*	*OAR2_55248792.1*- *OAR2_57832237.1*	*51658967*	*53837176*	*6.297*
	F_ST_-SS4	2	*OAR2_55493630_X.1*- *OAR2_57596413.1*	51898098	53597080	
	Churra-ObsHtz-SS5	2	*OAR2_55493630_X.1*- *OAR2_56828090.1*	51898098	52997998	
	*hapflk*-*SS1*	*2*	*OAR2_55493630_X.1*- *OAR2_56768579.1*	*51898098*	*52938537*	
	Churra-ObsHtz-SS6	2	*s18609.1*- *s53985.1*	53366034	53670410	
2	*XPEHH*-*SS7*	*2*	*OAR2_84010413.1*- *OAR2_84382185.1*	*78854385*	*79189919*	*4.571*
	F_ST_-SS5	2	*OAR2_84182215.1*- *OAR2_84382185.1*	79017511	79189919	
3	*XPEHH*-*SS13*	*3*	*s59799.1*- *OAR3_162871753.1*	*151088496*	*152334140*	*5.232*
	F_ST_-SS6	3	*OAR3_161831413.1*- *OAR3_162231144.1*	151512221	151778900	
	F_ST_-SS7	3	*OAR3_162782289.1*- *OAR3_162794870.1*	152215311	152227684	
4	*hapflk*-*SS4*	*3*	*OAR3_163071695_X.1*- *OAR3_164185125.1*	*152544998*	*153519437*	
	*XPEHH*-*SS14*	*3*	*s59746.1*- *OAR3_164185125.1*	*152644200*	*153519437*	*6.651*
	F_ST_-SS8	3	*OAR3_163342940.1*- *OAR3_163641518.1*	152795421	153090551	
	F_ST_-SS9	3	*OAR3_164115875.1*-*OAR3_164185125.1*	153459890	153519437	
5	*XPEHH*-*SS16*	*3*	*s26177.1*- *OAR3_165200988.1*	*154006814*	*154402834*	*4.324*
	F_ST_-SS10	3	*OAR3_164788310.1*-*OAR3_165324739.1*	154069702	154522600	
6	*XPEHH*-*SS17*	*3*	*OAR3_165450843.1*-*OAR3_169414477.1*	*154638280*	*158339544*	*5.409*
	F_ST_-SS11	3	*OAR3_166034748.1*- *OAR3_166122747.1*	155167107	155252399	
7	*XPEHH*-*SS24*	*3*	*s07782.1*- *s67950.1*	*179815920*	*180128893*	*4.066*
	Churra-ObsHtz-SS23	3	*OAR3_193567675.1*	179832455		
8	Churra-ObsHtz-SS24	3	*OAR3_196791000.1*- *OAR3_196913312.1*	182778735	182916410	
	*XPEHH*-*SS26*	*3*	*OAR3_196880003.1*- *OAR3_196904777.1*	*182867529*	*182900674*	*3.373*
9	Churra-ObsHtz-SS25	3	*s67036.1*- *OAR3_197402139.1*	183347210	183368930	
	Merino-ObsHtz-SS22	3	*OAR3_197402139.1*	183368930		
	*XPEHH*-*SS27*	*3*	*OAR3_197402139.1*- *OAR3_197466728.1*	*183368930*	*183429797*	*4.061*
10	*XPEHH*-*SS31*	*3*	*OAR3_201886269.1*- *OAR3_202943170.1*	*187634152*	*188481721*	*4.323*
	F_ST_-SS15	3	*OAR3_202741875.1*	188276666		
11	Merino-ObsHtz-SS35	6	*s73850.1*-*OAR6_40855809.1*	36461468	36655091	
	*XPEHH*-*SS43*	*6*	*s20660.1*- *s32980.1*	*36626596*	*36914376*	− ***4.211***
12	F_ST_-SS24	6	*s17946.1*	37164263		
	*XPEHH*-*SS44*	*6*	*s17946.1*- *OAR14_57922732.1*	*37164263*	*38580198*	− ***4.837***
	Merino-ObsHtz-SS36	6	*OAR6_42247197.1*	37987281		
	Merino-ObsHtz-SS37	6	*OAR6_42484920_X.1*	38214088		
	Merino-ObsHtz-SS38	6	*OAR6_42743614.1*- *OAR6_42834740.1*	38417881	38481174	
13	*XPEHH*-*SS55*	*8*	*s50528.1*- *OAR8_36294417_X.1*	*32778561*	*33477406*	*4.846*
	Churra-ObsHtz-SS37	8	*OAR8_35694056.1*- *OAR8_35827974.1*	32849509	32979538	
14	*XPEHH*-*SS60*	*8*	*OAR8_39847976.1*-*s27049.1*	*37075040*	*37422641*	*4.194*
	Churra-ObsHtz-SS38	8	*OAR8_39977285.1*- *OAR8_40079017.1*	37211967	37313171	
15	*XPEHH*-*SS62*	*10*	*OAR10_29381795.1*- *OAR10_29448537.1*	*29344224*	*29415140*	*3.716*
	F_ST_-SS29	10	*OAR10_29389966_X.1*-*OAR10_29737372.1*	29353089	29713193	
	Churra-ObsHtz-SS42	10	*OAR10_29511510.1*-*OAR10_29722772.1*	29476678	29688513	
16	Merino-ObsHtz-SS52	11	*OAR11_27752920.1*- *OAR11_28473036.1*	26512466	26939891	
	*XPEHH*-*SS77*	*11*	*s56248.1*-*s31301.1*	*26571629*	*26623188*	− ***3.459***
17	Merino-ObsHtz-SS70	15	*s19862.1*- *s00941.1*	74618189	74636302	
	XPEHH-SS95	15	*s19862.1*- *s00941.1*	74618189	74636302	− **3.429**
18	*Merino*-*ObsHtz*-*SS95*	*25*	*s30024.1*-*s67158.1*	*7356301*	*7727709*	
	F_ST_-SS49	25	*s31858.1*-*s44881.1*	7599609	7608913	
	*XPEHH*-*SS97*	*25*	*s44881.1*- *s74537.1*	*7608913*	*7821104*	− ***4.234***

After defining the selection signals identified by the different selection sweep mapping methods considered in our study, i.e. differentiation analysis (F_ST_-SS), identification of regions of reduced heterozygosity (ObsHtz-SS) and haplotype-based selection mapping methods hapFLK and XEHPP analyses (hapFLK-SS) and XEHPP-SS), the corresponding intervals were compared and Convergence Candidate regions (CCR) were defined when at least one haplotype-based method showed coincidence with any of the two other analyses performed

^a^Convergence candidate regions defined based on the convergence of selection signals identified in this study

^b^Selection signals identified by the four analysis methods used in this study: the methods based on the estimation of *F*_ST_ and observed heterozygosity (ObsHtz) and the two methods based on haplotype analysis (hapFLK and XPEHH). Note that the signals identified by the haplotype-based methods are indicated in italics. It was necessary that at least overlapping of one significant haplotype-based SS (identified by the hapFLK or the XPEHH analyses) and one SS identified by any of the two other methods (FST or ObsHtz-based analyses) to label a region as a CCR

^c^Chromosome

^d^For the SS identified with the XP-EHH test, the most extreme XP-EHH estimate is provided. Note that positive and negative (negative highlighted in bold font) estimates indicate selection in the Churra and Merino populations, respectively

Fifty-two annotated genes were extracted from the five Merino-defined CCR regions (see Additional file 13: Table S9), whereas 83 genes were extracted from the Churra-CCR intervals (see Additional file 14: Table S10). By comparing these genes with our database of reference candidate genes, 15 unique genes of interest were identified based on their known association with traits targeted by selection, such as horns (*RXFP2*), stature (*LCORL* and *NCAPG*), hair follicle cycle and wool quality (*IFNG*, *DVL2*, and *TP53*), meat production/quality traits (*TPM2*, *CACNG2*, *PVALB*, *ACADVL*, *SLC2A4*, *CHRNB1*, and *ATP1B2*), or dairy traits (*IFNG*, *ABCG2*, *TP53*, *SPP1*, and *DVL2*) (see Additional file 14: Table S11). We found that the five Merino-related CCR and the 15 Churra-related CCR overlapped, respectively, with 103 and 84 previously reported genetic QTL or associations with phenotypic traits (see Additional file 16: Table S12). For each defined CCR, the correspondence with previously reported selection sweeps is indicated in Table 2.

**Table 2:** Correspondence of the 18 convergence candidate regions (CCR) identified as putative selection signals for Churra and Australian Merino sheep populations with previously reported signatures of selection

Present study		Other studies		
Region	Genomic interval (Mb)	Correspondence with other studies Chr: peak marker/interval (Mb)	Putative candidate genes according to other studies	Population (target trait)
CCR1	Chr2: 51.659–53.837	OAR2: 52.266–52.454		Zel-Lori Bakhtiri and HapMap dataset [25] (fat deposition)
		OAR2: 52.40 (peak SNP)		HapMap dataset [21]
		OAR2: 51.41–53.44		HapMap dataset [22]
		OAR2: 51.72–51.95		HapMap dataset [28] (climate adaptation)
		OAR2: 51.200–52.100; 52.100–52.900; 53.60–54.5800		Duolang sheep [27] (ecoregion adaptation)
CCR2	Chr2: 78.854–79.190			
CCR3	Chr3: 151.088–152.393	OAR3: 150.5–154.2	*HMGA2, WIF1*	Spanish breeds [30]
		OAR3: 151.42–156.93	*HMGA2*	HapMap dataset [22]
		OAR3: 152.68–154.679		HapMap dataset [19] (dairy specialization)
CCR4	Chr3: 152.545–153.519	OAR3: 150.5–154.2	*HMGA2, WIF1*	Spanish breeds [30]
		OAR3: 152.68–154.679		HapMap dataset [19] (dairy specialization)
CCR5	Chr3: 154.007–154.523	OAR3: 154.213 (peak SNP)	*HMGA2, MSRB3, LEMD3*	HapMap dataset [21]
		OAR3: 154.79–154.93		HapMap dataset [22]
		OAR3: 151.42–156.93		HapMap dataset [22]
		OAR3: 150.5–154.2		Spanish breeds [30]
		OAR3: 152.68–154.679		HapMap dataset [19] (dairy specialization)
CCR6	Chr3: 154.638–158.339	OAR3: 154.79–154.93		HapMap dataset [22]
CCR7	Chr3: 179.816–180.129			
CCR8	Chr3: 182.779–182.916	OAR3: 182.00–184.00		Duolang sheep [27] (ecoregion adaptation)
CCR9	Chr3: 183.347–183.430	OAR3: 182.00–184.00		Duolang sheep [27] (ecoregion adaptation)
CCR10	Chr3: 187.634–188.482			
CCR11	Chr6: 36.461–36.914	OAR6: 36.073 (peak SNP)	*ABCG2, NCAPG, PDK2*	HapMap dataset [21]
		OAR6: 34.71–39.12		HapMap dataset [22]
		OAR6: 36.63–36.8		HapMap dataset [28] (climate adaptation)
		OAR6: 36.200–36.500		Duolang sheep [27] (ecoregion adaptation
		OAR6: 30.367–41.863		HapMap dataset [19] (dairy specialization)
CCR12	Chr6: 37.164–38.580	OAR6: 34.71–39.12		
		OAR6: 37.2–38.0		HapMap dataset [22]
		OAR6: 37.40–37.60		HapMap dataset [24]
			*LCORL, NCAPG*	Small-tailed Han sheep [27] (ecoregion adaptation)
		OAR6: 30.367–41.863		HapMap dataset [19] (dairy specialization)
CCR13	Chr8: 32.779–33.477	OAR8: 32.159 (Peak SNP)	*BVES*	HapMap dataset [21]
CCR14	Chr8: 37.075–37.423			
CCR15	Chr10: 29.344–29.713	OAR10: 29.476 (peak SNP)	*RXFP2*	HapMap dataset [21]
		OAR10: 29.1–29.3		Spanish breeds [30]
		OAR10: 28.50–30.50		HapMap dataset [22]
		OAR10: 28.71–29.00		HapMap dataset [28] (climate adaptation)
				HapMap dataset [24]
				HapMap dataset [24]
		OAR10: 27.1–31.2		Small-tailed Han sheep [27] (ecoregion adaptation
		OAR10: 29.1–31.9	*RXFP2, B3GALTL*	Duolang sheep [27] (ecoregion adaptation)
		OAR10: 29.40–29.700		
		OAR10: 29.50–29.400		
CCR16	Chr11: 26.512–26.940	OAR11: 24.18–38.74		HapMap dataset [22]
		OAR11: 26.8–29.9		Barki sheep versus temperate breeds (hot arid environment) [29]
				Small-tailed Han sheep [27] (ecoregion adaptation)
CCR17	Chr15: 74.618–74.636	OAR15: 72.774–74.55		HapMap dataset [19] (dairy specialization)
CCR18	Chr25: 7.356–7.821	OAR25: 7.517 (peak SNP)		HapMap dataset [21]
		OAR25: 7.400–7.600		Duolang sheep [27] (ecoregion adaptation)

### Selection mapping validation results

The results from the individual analyses performed for the samples included in the validation analysis are in Additional file 17: Table S13 and graphically represented in Additional file 18: Figures S5 and S6. The genetic differentiation analysis identified 39 candidate selection sweeps, whereas the scans looking for regions of reduced observed heterozygosity identified 97 and 68 candidate selection sweeps for Churra and Spanish Merino, respectively. The *hapflk* and the XP-EHH analyses identified, respectively, six and 76 significant genomic regions as potential selection signatures. Based on the selection signals identified in these individual analyses, and applying the same overlapping requirements than in the core analyses, we defined 18 CCR (labelled as CCR101 to CCR118, as shown in Additional file 19: Table S14). The correspondence between these CCR and those identified in the core analyses are also indicated in Table 3. In summary, seven of the Spanish Merino-Churra CCR were directly related with six of the CCR identified between Australian Merino and Churra breeds (those highlighted in blue), although some others were close to a previously identified CCR (e.g. CCR109 and CCR110 could be considered also related to CCR13). The core CCR that were clearly validated by this secondary analysis were CCR1, CCR3, CCR4 and CCR13 for Churra and CCR12 and CCR28 for Australian Merino. These validated CCR were, in general, those showing the most extreme XP-EHH estimates in the core analyses (e.g. all of them had an absolute XP-EHH estimate higher than 4.80, with the exception of CCR18, located on OAR25, which had an estimate of − 4.233).

**Table 3:** Correspondence between the convergence candidate regions (CCR) identified in the core analyses between Australian Merino and Churra breeds (labeled as CCR1 to CCR18), with the CCR identified in the validation analyses performed by contrasting a small dataset of Spanish Merino and Churra sheep genotypes (labeled as CCR101 to CCR118)

CCR AustralianMerino-Churra			CCR SpanishMerino-Churra		
Region	Genomic region	Most extreme XPEHH value^a^	Region	Genomic region	Most extreme XPEHH value^a^
CCR1	Chr2: 51.659–53.837	6.297	CCR101	Chr2: 51.530–53.798	4.282
CCR2	Chr2: 78.854–79.190	4.571			
CCR3	Chr3: 151.088–152.393	5.232	CCR102	Chr3: 151.433–152.055	3.648
CCR4	Chr3: 152.545–153.519	6.651	CCR103	Chr3: 152.855–152.861	3.560
CCR5	Chr3: 154.007–154.523	4.324			
CCR6	Chr3: 154.638–158.339	5.409			
CCR7	Chr3: 179.816–180.129	4.066			
CCR8	Chr3: 182.779–182.916	3.373			
CCR9	Chr3: 183.347–183.430	4.061			
CCR10	Chr3: 187.634–188.482	4.323			
			CCR104	Chr4: 30.499–30.929	− **4.131**
CCR11	Chr6: 36.461–36.914	− **4.211**			
CCR12	Chr6: 37.164–38.580	− **4.837**	CCR105	Chr6: 38.181–38.255	− **3.666**
			CCR106	Chr6: 38.429–38.617	− **4.256**
			CCR107	Chr8: 31.613–31.699	
CCR13	Chr8: 32.779–33.477	4.846	CCR108	Chr8: 32.364–32.597	
			CCR109	Chr8: 33.676–34.622	
			CCR110	Chr8: 34.791–35.740	
CCR14	Chr8: 37.075–37.423	4.194			
			CCR111	Chr8: 51.730–52.676	− **5.015**
			CCR112	Chr8: 52.997–54.352	− **4.599**
			CCR113	Chr8: 59.193–60.187	− **6.377**
CCR15	Chr10: 29.344–29.713	3.716			
			CCR114	Chr10: 51.490–52.154	− **4.592**
			CCR115	Chr10: 52.389–52.670	− **3.590**
CCR16	Chr11: 26.512–26.940	− **3.458**			
			CCR116	Chr15_ 37.553–37.776	4.543
			CCR117	Chr15: 38.783–38.943	3.734
CCR17	Chr15: 74.618–74.636	− **3.429**			
CCR18	Chr25: 7.356–7.821	− **4.234**	CCR118	Chr25: 7.356–7.970	− **3.361**

^a^For the CCR including a selection signal identified by the XP-EHH test, the most extreme XP-EHH estimate is provided. Positive and negative (highlighted in bold font) XP-EHH estimates indicate selection in the Churra and Merino populations, respectively

### Identification and annotation of divergent allelic variants in the identified CCR based on the analysis of whole-genome sequencing data

#### Results of the variant calling analysis

The maximum length of the reads obtained through the sequencing process was 100 bp. The whole-genome sequence datasets showed an average number of raw reads per sample of 318,377,494 paired reads. We obtained an average of 296,228,613 reads per sample that passed the quality control process. Per sample, the number of reads aligned to the reference genome varied between 112,607,669 and 513,347,097, with an average of 293,341,790 and an average of 2% unmapped reads per sample. The number of duplicates per sample ranged from 4,818,140 to 43,064,209, with an average of 16,747,357. After alignment, focusing on the 18 target CCR intervals, the individual analyses with GATK and Samtools identified, initially, 194,413 and 196,128 genetic variants respectively (175,317 and 174,278, respectively, after applying the Snpshift filters). The intersection between the variants identified by the two different software programs showed 142,400 variants commonly identified for the 28 sheep genomes analyzed. All these variants, which included 139,745 bi-allelic SNPs and 2655 indels, were considered for further analyses.

#### Identification of the divergent SNPs in the CCR regions

**Fig. 4:**
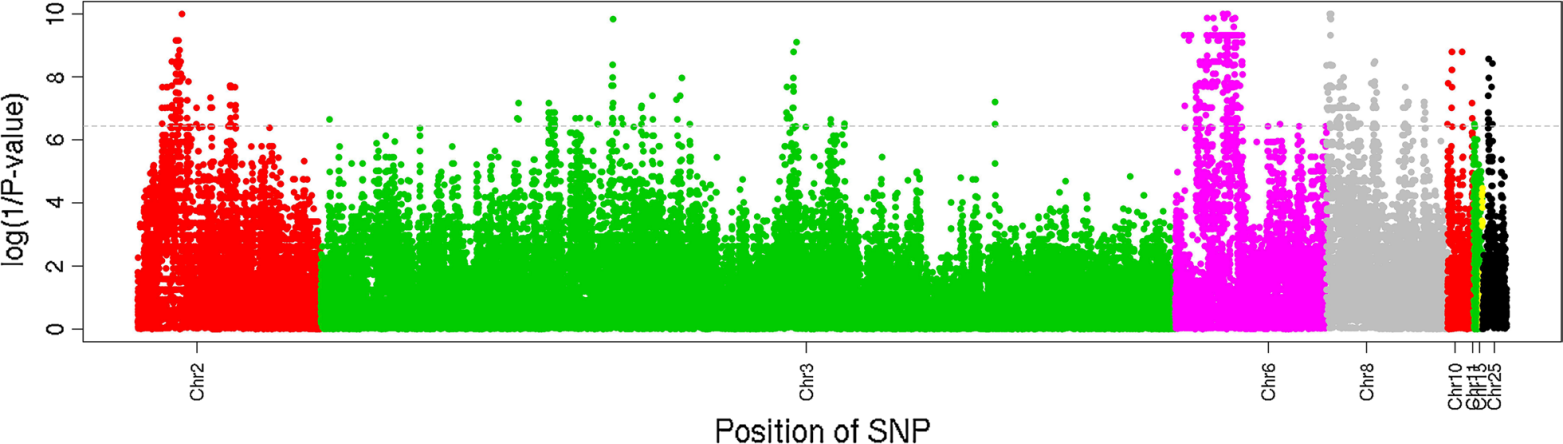
Results of the association analysis performed for the 135,061 SNPs from the processing of 28 whole-genome sequencing samples of Churra and Australian Merino sheep breeds with the aim of identifying the markers with the most divergent allele frequencies between the two breeds compared. Genome-wide distribution of the log (1/*P* value) obtained from the association analysis with the breed identity are represented on the Y-axis. The horizontal line represents the significance threshold considered after a Bonferroni correction for multiple testing (*P* < 0.05/139,244 = *P* value < 0.000000359; log (1/*P* value) = 6.44)

All 28 samples and 139,244 variants, including SNPs and indels, passed the genotype QC filtering steps and were subsequently investigated by association analysis to compare Australian Merino and Churra breeds. Among these variants, 25,774 do not have an associated *rs* number. The results of this analysis for the tested SNPs are represented graphically in Fig. 3, where the X-axis shows the log (1/*P* value). Following a Bonferroni correction for the number of variants analyzed, 1291 variants (1282 SNPs and 9 indels) exceeded the 5% experiment-wise significance threshold (*P* < 0.05/139,244 = *P* value < 0.000000359; log (1/*P* value) = 6.44). The distribution of these divergent variants over the considered chromosomes was as follows: 216 variants on OAR2, 117 on OAR3, 593 on OAR6, 316 on OAR8, 17 on OAR10, 2 on OAR11, 30 on OAR25, and none on OAR15 (Fig. 4). Considering the level of difference in allele frequencies (D) between the two breeds analyzed, which are in Additional file 20: Table S15, differences higher than 0.7 were shown by 79 variants (78 SNPs and 1 Indel) with a high frequency in Churra. Among them, the most extreme value of divergent allele frequency (D = − 0.8) were one SNP on OAR3 (*rs408539160*) and seven on OAR10 located in the interval 29.380–29.499 Mb within the *EEF1A1* gene (*ENSOARG00000011616*), with the exception of *rs421531355*, which is an intronic variant of the *RXFP2* gene. For the variants with a high frequency in Merino and low in Churra, 943 showed D values higher than 0.7; the most extreme of these D values were found for variants located on OAR3, 6 and 8 (see Additional file 20: Table S15).

The results of the functional annotation analysis showed that 1291 divergent variants included 257 intragenic variants causing 296 annotation variants, distributed across 31 protein coding genes, two pseudogenes (one of them identified as orthologous of bovine *TPI1*), one rRNA (*5S_rRNA*) and two snRNA (see Additional file 21: Table S16). Note that all the significant variants identified in the studied region of OAR25 were located in intergenic regions. The genetic variations included in protein coding genes resulted in 275 functional annotation variants classified as four missense variants, four synonymous variants, 199 intronic variants, 38 upstream gene variants, 29 downstream gene variants, and one variant in a 3′ UTR region.

Focusing on the variants located in exons of the above mentioned genes (see Additional file 21: Table S16), all of which were SNPs, we found the following: (1) on OAR2, a synonymous variant in the *PHF24* gene and a missense mutation in the *NPR2* gene, (2) on OAR6, three synonymous variants, in *PDK2*, *FAM184B* and *ENSOARG00000004249* (orthologous to human *LCORL* by *BLASTN*), and three missense variants, in *NCAPG* and the inferred *LCORL* gene.

A full characterization of the identified missense variants is in Table 4. This is based on the eVEP annotation for the *NPR2* and *NCPAG* genes. For a proper assessment of the effects of the *LCORL* missense mutations, we aligned the interval including the two mutations identified in this gene against the most updated version of the sheep reference genome Oar_v4.0 [76] using a BLASTN search. Then, we identified the effect of the two *LCORL* missense mutations in the mRNA gene sequence (XM_015096407.1) and the corresponding protein sequence (XP_014951893.1; ligand-dependent nuclear receptor corepressor-like protein isoform X1). The prediction of the functional impact of the amino-acid changes of these mutations with the SIFT software [75] considered the mutations in the *NPR2* and *LCORL* genes as “tolerated”, whereas the missense mutation located in the *NCAPG* gene, *NCAPG_Ser585Phe*, was classified as “deleterious” (score = 0.0) (Table 4).

**Table 4:** Characterization of the three missense mutations identified in this study

Features	Missense mutations identified as divergent variants based on the analysis of whole-genome sequence datasets from Churra and Australian Merino samples			
SNP position (Oar_v3.1)	52,429,848	37,308,727	37,3557,21	37,356,400
Chromosome	2	6	6	6
dbSNP_ID	*rs160159505*	*rs159958168*	*rs419074913*	*rs159958380*
Gene	*NPR2*	*NCAPG*	*LCORL* ^a^	*LCORL* ^a^
Ref. (Texel Oar_v3.1) → Alt^b^	T → C	C → T	T → A	A → T
Position in CDS	c.2540	c.1754	c.4321^c^	c.3642^c^
Base pair substitution in CDS	T → C	C → T	A → T	T → A
Breed (mutant allele)^d^	Merino	Merino	Churra	Merino
Codon change	cAc → cGc	TCC → TTC	ATA → TTA	GAT → GAA
Amino acid change	Histitine (H) → Arginine (R)	Serine (S) → Phenilalanine (F)	Isoleucine (I) → Leucine (L)	Aspartate (D) → Glutamate (E)
Protein change	*NPR2_His847Arg*	*NCAPG_Ser585Phe*	*LCORL_Ile1441Leu*	*LCORL_Asp1214Glu*
Functional impact (ensemblVEP_Oarv3.1)	Moderate	Moderate	Moderate	Moderate
Functional impact (Polyphen-2)	Benign	Benign (score = 0.252; sensitivity: 0.91; specificity: 0.88)	Benign	Benign
Functional impact (SIFT_Oarv3.1)	Tolerated	Deleterious	Tolerated (low confidence)	Tolerated
Properties of wild aminoacid	Moderate hydropathy, charge “+”	Hydrophilic, polar, no charge	Hydrophobic, no charge	Hydrophilic, charge “−”
Properties of mutant aminoacid	Hydrophilic, charge “+”	Hydrophobic, apolar, no charge	Hydrophobic, no charge	Hydrophilic,, charge “−”
Churra genotypes	TT (15)	CC (14), CT (1)	AT (1), TT (14)	AA (14), AT (1)
Australian Merino genotypes	CC (9), TC (4)	TT (9), TC (3), CC (1)	AA (9), AT (3), TT (1)	TT (9), TA (3), AA (1)

^a^Mutation initially annotated within the *ENSOARG00000004249* novel gene (Oar_3.1). BLASTN analyses showed correspondence with the human *LCORL* gene and the ovine *LCOR* according to the most recent version of the sheep genome (Oar_v4.0)

^b^Ref. (Texel Oar_v3.1) → Alt: Reference and alternative alleles, respectively, identified in the analysis of the whole-genome sequence datasets

^c^Position of the SNP in the coding sequence based on the alignment of the sequence harboring the mutation to the annotation of the *LCORL* gene in the most recent version of the sheep genome (Oar_v4.0): NCBI Reference sequences: XM_015096407.1, XP_014951893.1 (ligand-dependent nuclear receptor corepressor-like protein isoform X1)

^d^Breed with the highest frequency for the mutant allele (regarding the wild protein sequence). Note that for SNP *rs419074913*, the Texel sheep of the reference genome harbors the mutant allele according the CDS and protein sequence

## Discussion

Based on the increasing affordable cost of next-generation sequencing technologies [77], the information derived from whole-genome resequencing offers increased detection power and a higher resolution to identify the genetic variants that underlie variation in traits of economic interest or linked to selection events in livestock populations [78–80]. In this work, we exploited the information from WGSeq datasets as a high-resolution step to investigate regions that were previously identified through the analysis of medium-density SNP panels in a representative sample of the population(s).

### Identification of selection sweeps in Australian Merino and Churra sheep breeds

The putative selection sweep regions (referred herein as CCR) were determined by comparing 50 K-chip genotypes of three Australian Merino strains that are highly specialized for wool production with genotypes of the related, coarse-wool Spanish Churra dairy breed. In agreement with other authors [21, 22], our population structure analysis supported the use of these two groups of samples as appropriate for mapping selection sweeps because of their close phylogenic relationship but divergent phenotypic characteristics (see Additional file 1: Table S1). The contrasting features of the two breeds [e.g. white and fine wool, growth/carcass production, parasite resistance selection of Australian Merino; coarse wool, milk production/composition, dairy udder/body conformation, and characteristic black patches in specific body regions of Churra; (see Additional file 1: Table S1)] may help to identify the phenotypic targets of putative selection sweeps. Considering the possibility that the geographical isolation and distance between the two studied populations could be a confounding effect with respect of the signals evidenced we have, in addition, performed a validation analysis by contrasting an available dataset of Spanish Merino genotypes with the Churra sheep breed.

In addition, the high genetic diversity reported for the contrasting breeds [30, 81, 82] should be taken into account, which fits well with the known history of these breeds. In particular, the Australian Merinos have been reported as the most diverse sheep populations [81, 83, 84] since the foundation of this population involves contributions from different European, Asian and African breeds and, therefore, Australian Merino are a combination of strains of sheep rather than a single, ancient, homogenous breed. The Australian Merino lines considered in this study have some of the highest estimates for effective population size at 50 generations ago (average of *Ne* = 868; assuming four years per generation) [21]. The first historical references about Churra sheep date from the Middle Ages, approximately 800 years ago [85]. This breed shows a large influence from ovine populations brought in the Iberia Peninsula by the Celts [86]. Compared with other breeds, the estimated *Ne* at 50 generations ago for Churra is intermediate (*Ne* = 600) [21], and a steady decrease of this value until the start of the breeding program in 1986 suggests the absence of severe bottlenecks or other extreme demographic events [82, 87]. Selection sweep mapping methods that are based on haplotypes are known to be highly robust towards perturbations of the demographic model when compared with methods based on population subdivision or allele frequencies [9]. Hence, in our work, the labelling requirement of overlap between at least one haplotype-based method and the *F*_ST_/ObsHtz methods may be seen as helping to limit the number of false positive results due to neutral (e.g. demographic) processes.

Comparing the results of the different analyses, we found discrepancies in the number of signals detected. For example, the hapfFLK analysis, which accounts for the relationship between populations and the LD pattern (haplotype diversity), only detected seven candidate signatures of selection, compared with 25 detected by the genetic differentiation approach, 96 and 77 regions of signatures of selection of reduced heterozygosity in Australian Merino and Churra, respectively, and 98 regions identified by the XP-EHH analysis. These differences can be explained by the fact that the different statistics used in selection sweep mapping do not capture the same patterns in the data, as previously pointed out by many authors [23, 88, 89]. The small number of significant regions detected with hapFLK compared with the other methods agrees with other studies that have analyzed the same datasets using hapFLK and other methods [30, 90]. As suggested by Manunza et al. [30], the multipoint linkage LD model implemented by hapFLK may explain the higher stringency of this method when compared with methods that do not consider haplotype structure (such as *F*_ST_ or ObsHtz). In contrast, XP-EHH analysis, which also makes use of haplotype information, detected a large number of selection sweeps (98) supporting several of the signatures of selection detected by the ObsHtz and *F*_ST_ analyses. Hence, the underlying model of this cross-population analysis for which the basic idea is to test if each site is homozygous in one population and polymorphic in the other population appears to fit efficiently for the Churra versus Australian Merino contrast undertaken in our study. Also the ObsHtz analysis identified a substantial number of candidate regions (96 Merino/77 Churra), which agrees with the fact that signatures of selection that are based on a reduction in genetic diversity persist for a longer period of time than signals based on haplotype structure and thus the former can detect older signatures of selection [88, 89]. The intermediate number of candidate regions detected based on population differentiation agrees with the intermediate position suggested for these methods by Sabeti et al. [88] regarding the time scale persistency for the different selection sweep mapping methods.

Similar discrepancies in the number of candidate selection sweeps identified by the different methods in the Spanish Merino and Churra analyses (39 with *F*_ST_, 97 for ObsHtz-Churra, 68 for ObsHtz-SpanishMerino, 7 for hapflk and 76 with XP-EHH) (see Additional file 17: Table S13) prove that the differences observed in the number of signatures of selection in the Australian Merino versus Churra analyses were not due to the confounding effect of genetic drift and geographical isolation of the breeds analyzed. The fact that the six clearly confirmed CCR were those that had the most extreme XP-EHH estimates in the Australian Merino-Churra core analyses appears to suggest that the lack of confirmation of some other regions (e.g. CCR2 on OAR2, CCR16 on OAR11, CCR17 on OAR15) can be related to the lower power of detection of the validation analyses due to the limited number of samples analyzed (20 Spanish Merino and 20 Churra samples). Overall, we think that the validation strategy presented here supports the validity of the selection sweeps identified when contrasting Australian Merino and Churra sheep.

In addition, the combination of the four methods used in this work provides a comprehensive picture of the different types of selection sweeps present in the genomes of Churra and Australian Merino sheep. The requirement of overlap between regions identified by different methods to define a selection sweep increases the reliability of the 18 CCR reported here to result from genuine selection events. This is supported by the high level of positional correspondence of these regions with selection sweeps previously reported in sheep (Table 2).

### Exploration of convergence candidate regions through WGSeq

In this study, we exploited WGSeq as a secondary step to provide a detailed study of the genetic variation within the regions previously identified as potential signatures of selection. As a technical issue and considering that paired-end sequencing is preferred over single-end sequencing, since it allows improved identification of duplicated reads and a better estimation of the fragment size distribution [91], it is worth clarifying that the workflow used in this study was based on Trimmomatic, which provides a flexible method to keep, in the analysis, the reads for which their paired read is filtered during the quality control filtering [66]. Also the reads for which their paired read was unmapped were included in the later variant calling analysis. To assess the impact that the use of singletons could have on the results, we repeated the variant calling analysis without considering singletons, and found a concordance level of 99.15%. This observation suggests that, for the variant calling analysis workflow applied in this study, which considers the common variants identified by GATK and Samtools, the use of singletons does not have a negative impact on the quality of the variant calling analysis; however, we can also consider than using a simpler workflow that eliminates these singletons may be an efficient strategy for future studies.

#### Merino-related convergence candidate regions

Among the genomic regions showing positive selection in Merino, those located on OAR6, CCR11 (36.641–36.914 Mb) and CCR12 (37.164–38.580 Mb) showed substantial overlap with previously reported selection sweeps (Table 2) and QTL in sheep (see Additional file 16: Table S12). While most of the coincidences with selection sweeps reported in these regions are related to studies on the SheepHapMap dataset (Table 2), the study of Liu et al. [27] on adaptation to different ecoregions (regions where sheep are exposed to different climate, environment and feeding conditions) also identified the region OAR6: 36.200–36.500 Mb as a putative signature of selection in Duolang sheep. Furthermore, a large number of QTL/associations with production traits have been mapped within these two CCR in a population of Scottish Blackface lambs [92] (see Additional file 16: Table S12). Several of those effects were associated with carcass bone percentage and fat carcass traits, with some suggestion of muscle density effects. CCR11 and CCR12 also include QTL for growth traits [93, 94] and birth weight [95]. Most of these studies suggest *NCAPG* and *LCORL* as strong candidate genes for these effects, due to the reported associations of these loci with human stature [96] and body size in mammals [97–102]. In addition to these sheep studies, selection sweeps have been identified around the *NCAPG*-*LCORL* locus in dogs and pigs [49, 103].

In many cases, the intervals flanking the previously reported selection sweeps or the QTL reported in this OAR6 genomic region involve both CCR11 and CCR12. Considering the inherent inaccuracy of gene mapping, even when based on medium-density SNP arrays, and the large number of effects identified in that region, our approach to group individual signatures of selection based on the extent of LD of the studied breeds appears to identify two independent selection sweeps, which may help to differentiate the causal mutations that underlie the various QTL effects reported in this genomic region.

CCR11 contains the following annotated genes: *ABCG2*, *PKD2*, *SPP1*, *MEPE*, and *IBSP* (see Additional file 21: Table S16). Our high-resolution analysis based on sequence data showed that the CCR11 SNPs showing a significant association with breed identity between Churra and Australian Merino were located in three genes included in that interval: *PKD2*, *MEPE* and *IBSP*. Three intronic and one synonymous divergent variant were identified in the *PKD2* gene. In cattle, one SNP within this gene is significantly associated with hot carcass weight and intermuscular fat percentage [104]. The other divergent variants were located in non-coding regions of the *MEPE* and *IBSP* genes. *MEPE* is thought to play an inhibitory role in bone formation, and disruption of one of its alleles is known to increase bone mass in mouse [105]. No significant associations were identified with markers within *ABCG2* and *SPP1* which are functional candidate genes for milk production traits [106, 107].

**Fig. 5:**
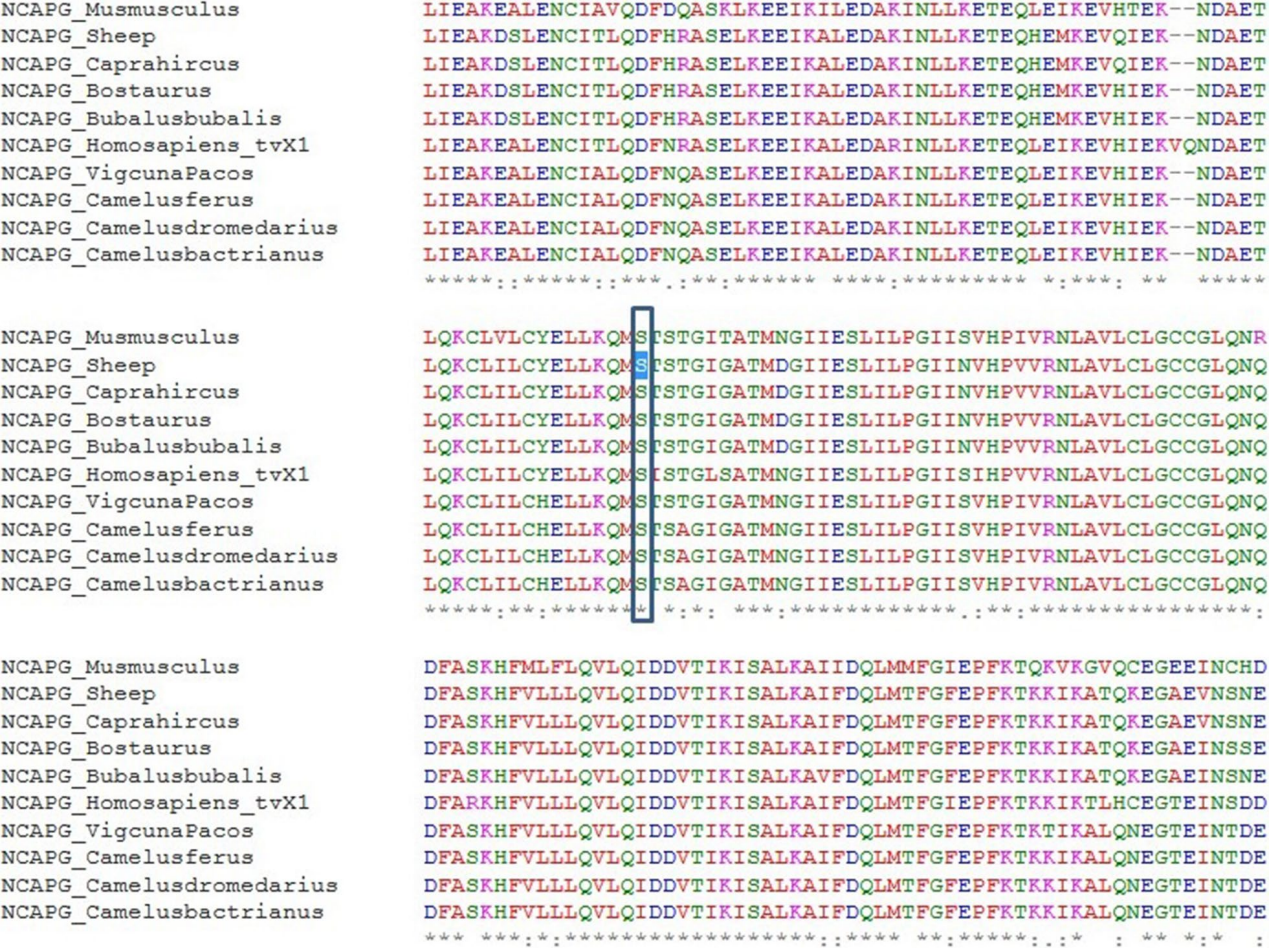
Multi-species alignment of the amino-acid sequence of the NCAPG protein across ruminant species, humans and mouse using Clustal Omega (http://www.ebi.ac.uk/Tools/msa/clustalo/). The serine amino acid affected by the *NCAPG_c.1754C* > *T* mutation shows a high conservation level in all species considered

CCR12 (OAR6: 37.164–38.580 Mb) includes four annotated genes, the major candidate genes *NCAPG* and *LCORL*, as well as *FAM184B*, which is highly expressed in skeletal muscle (http://www.proteinatlas.org/ENSG00000047662-FAM184B/tissue), and *DCAF16*. A considerable proportion of the intragenic variants (88/296) that show significant between-breed divergence were located within these four genes (see Additional file 21: Table S16), including two synonymous variants in *LCORL* and *FM184B* and three missense mutations in *NCAPG* and *LCORL*. Kühn et al. [108] suggested that the mechanism underlying the association between *NCAPG* and pre- and post-natal growth in several mammalian species may be related to the role of this gene in the modulation of growth and body tissue deposition by indirect effects on the nitric oxide (NO) pathway. In cattle, *NCAPG* is also suggested to be involved in early muscle development. [109]. An analysis in UniProt [110] shows the *NCAPG_Ser585Phe* amino acid substitution to affect the C-terminal, cysteine-rich domain of the protein, whereas a high level of across species conservation for this residue of the NCAPG protein (Fig. 5) was shown in a comparative analysis with Clustal Omega (http://www.ebi.ac.uk/Tools/msa/clustalo/). Further functional studies are needed to assess how this mutation may affect the protein function and possible effects on phenotype(s). The other gene for which divergent missense mutations were identified in this region is *LCORL*, which encodes a transcription factor that binds specific DNA elements and appears to function in spermatogenesis; polymorphisms in this gene have also been associated with skeletal frame size and human height, as previously discussed.

The results of the sequence analysis in our study support the hypothesis that the *NCAPG*-*LCORL* locus may be responsible for direct effects on sheep production traits, possibly including growth and/or carcass traits, which differ between Churra and Australian Merino. Studies that compared carcass characteristics of Churra and Spanish Merino at the same slaughter age showed that Merino has significantly higher hot carcass weight and conformation score than Churra [111], and also higher carcass yield, total muscle and bone percentage [112]. Hence, the three missense mutations identified within the *NCAPG* and *LCORL* genes by our high-resolution study should be further studied to assess their potential role as causal mutations of the previously reported QTL effects within that genomic region (e.g. Matika et al. [92]). This is the first study that suggests specific mutations in this region that may influence production traits in sheep. As the single “deleterious” mutation identified in this region, the *NCAPG_Ser585Phe* protein variant should be considered as the top candidate to explain the CCR12 signature of selection or the potential selection sweep nucleotide (SSN). Analysis of this mutation in meat breeds where the mutation segregates will allow the verification of the true effect of this polymorphism on production traits. In order to obtain further information about the potential link between *NCAPG* and *LCORL* missense mutations and meat production traits, we extracted the genotypes for these three polymorphisms from the 453 samples analyzed in the “PRJEB14685” Project of the EVA repository (http://www.ebi.ac.uk/ena/data/view/PRJEB14685). As observed from these genotypes (see Additional file 22: Table S17), the Merino-related allele for the three mutations, is only present (in homozygous or heterozygous status) in “meat production” breeds.

Three other Merino-CCR regions, CCR16, CCR17 and CCR18, are located on OAR11, 15 and 25, respectively. CCR16 (OAR11) is the most interesting, from which six of the 42 annotated genes were highlighted by the survey performed against our database of candidate genes (see Additional file 15: Table S11). Among them, *TP53* and *DVL2*, are both candidates for wool production due to their link to the hair follicle cycle [45]. However, the divergent intragenic variants identified in this region did not affect any of these wool-related candidate genes but included non-exonic variants within the *DLG4* and *ACADVL* genes. The *ACADVL* gene encodes the enzyme that catalyzes the first step of the mitochondrial fatty acid beta-oxidation pathway, which suggests that there may be differences in the fatty acid composition between Churra and Merino lamb meat.

A large proportion (7/9) of the previously reported QTL located within CCR18 (OAR25: 7.356–7.821 Mb) are wool-related QTL (see Additional file 16: Table S12). The study of Allain et al. [113], based on microsatellite markers, suggested the presence in this region of a locus with a major effect on fleece characteristics. These results were supported by another study based on the 50 K-chip in which genome-wise significant SNP associations for seven wool-related traits were reported in a nearby interval (OAR25: 6.1–8.2 Mb). Interestingly, a 2-kb insertion was identified at this location, which is a potential causal mutation for the absence of long, coarse hair in the birthcoat of the Romane breed [114]. This is a trait that is moderately genetically correlated with wool quality traits, with the woollier lambs showing a lower coefficient of variation, fewer fibers thicker than 30 μm and better wool quality compared with the other hairier lambs [115]. Within CCR18, 30 SNPs showed a significant difference in allele frequencies between Churra and Merino populations, although none of these SNPs were intragenic. Among the genes found in this region (see Additional file 13: Table S9), the novel gene *ENSOARG00000017989* shows correspondence with the bovine *EIF2S2* gene, which encodes the beta subunit of EIF-2 that functions in the early steps of protein synthesis. *EIF2S2* has been suggested as a novel candidate gene in relation to skin color in humans [116]. Because white wool color has been a major selection objective in Australian Merino, a possible link between the *EIF2S2* gene and the CCR18 Merino effect of the signature of selection should be investigated further.

#### Churra-related convergence candidate regions

With regard to the 15 Churra-associated CCR, nine overlapped with previously reported selection sweeps in sheep (Table 2). The region that showed correspondence with the largest number of studies (most based on SheepHapMap data) is CCR15 (OAR10: 29.334–29.713 Mb). This region includes the *RXFP2* gene, which is suggested to control the presence and size of horns in wild and domestic populations of sheep [21, 79, 117] and to be important for horn development in goats and cattle [118, 119]. *RXFP2* is a receptor for the relaxin and insulin-like factor 3 proteins and its effects on horn size and status appear to depend on its biochemical interactions with testosterone effects [120, 121]. This region was identified by XP-EHH and ObsHz analyses and overlapped with a signal from the *F*_ST_ analysis, which suggests positive selection in Churra (Table 1). Taking into account that selection for the polled phenotype has occurred in both Churra and all Australian Merino lines (not only the Australian Poll Merino), the detection of CCR15 as a Churra-related selection sweep region suggests that selection for the absence of horns is more recent (and thus more detectable) in Churra than in Merino. In fact, the high selection pressure for polledness in Churra started with the breeding program in 1985 [39], and prior to that time horned rams were preferred by Churra breeders (F. de la Fuente, personal communication). At present, about 90% of the Churra males are polled. There are practically no females with horns, and when horns are present they are rudimentary. For the OAR10 selection sweep, our high-resolution analysis identified 13 significant divergent intronic variants in the gene and one variant in the third intron of the *RXFP2* gene. A 1.8-kb insertion reported in the 3’ UTR region of the ovine *RXFP2* gene, which includes two exons of the *EEF1A1* gene, has been suggested to explain the polled phenotype in some sheep breeds [122]. However, this insertion does not completely segregate with the horn status in breeds with a variable horn status in both sexes or with a sex-dependent horn status [123], which is the horn status in Churra sheep. An additional exploratory association analysis considering only the Churra and Australian Poll Merino SNPs located on OAR10 (5329 polymorphic SNPs) only identified one significant SNP, which was annotated as an intronic variant within the *EEF1A1* gene (at 29.380.801 bp; *P* value = 0.000006769). Additional research based on whole-genome sequencing data from breeds with a variable horn status may shed light on this complex phenotype.

Two of the other Churra-related CCR, CCR1 (OAR2: 51.659–53.837) and CCR4 (OAR3: 152.545–153.519), were the only CCR supported by the hapFLK analysis, which identified the smallest number of regions in our analyses. These two regions, which were also replicated when analyzing Spanish Merino and Churra genotypes, overlap with 11 and seven QTL, respectively, reported for a range of sheep production traits (see Additional file 16: Table S12). The CCR1 region on OAR2 was the only one detected by all four selection sweep mapping methods (Table 1) and overlaps with several previously reported signatures of selection (Table 2). The intragenic significant divergent variation identified in this region included a missense mutation in the *NPR2* gene, *rs160159505* (OAR2: 52,429,848 pb). This gene was previously suggested by other authors [21, 22] as a strong candidate gene for ovine selection sweep effects reported in this region due to its major role in the regulation of skeletal growth regulation [124]. In humans, mutations in this gene are related to impaired skeletal growth [124, 125]. Because *rs160159505* was the mutation showing the highest significant association with breed identity within CCR1 (*P* value = 0.0000000001006) and although it was classified as “tolerated”, further studies should assess the possible relationship of this SNP with growth- or size-related traits in sheep. CCR4, the other region identified by hapFLK, showed correspondence with a signature of selection that was previously reported in a study on ovine signatures of selection related to dairy specialization (also including the Churra breed) [19]. The *LALBA* (OAR3:137.390–137.392) gene, which was suggested to harbor a quantitative trait nucleotide (QTN) for QTL effects related to milk composition traits in Churra sheep [126], maps just outside the boundaries of this region. The divergent intragenic variants annotated within this region were in non-coding regions of the genes *HELB* and *IRAK3,* which were not highlighted by our candidate gene survey. The other Churra-related CCR are not discussed in detail, but we would like to mention that CCR3 (OAR3: 151.088–152.393 Mb) overlaps with three QTL for resistance to gastrointestinal infection (see Additional file 16: Table S12) and that our association analyses identified, within this interval, a single significant intergenic SNP (*rs421227322*) located in the upstream region containing three immune-related genes, *IL22*, *IL26* and *IFNG*. Also of possible relevance is the identification of three intronic variants within the well-defined region of CCR5 (OAR3:154.006–154.522 Mb), which show extreme allele frequencies between Churra and Merino within the genes *MSRB3* and *LEMD3*. Indeed, intronic variation within these two genes has been directly associated with a pleiotropic QTL reported in cattle for birth weight, calving ease direct, marbling and ribeye muscle area [127].

## Conclusions

We have identified 18 putative selection sweeps by contrasting the Australian Merino and Spanish Churra sheep breeds. The phenotypes affected by these genetic effects may involve any trait for which selection was implemented in only one of the two compared breeds. The criteria used to define the selection candidate regions based on multiple mapping methods, together with a validation approach based on a comparison between Spanish Merino and Churra genotypes, support the validity of our mapping results and confirm the value of using selection mapping to detect genomic regions that influence the phenotypic variation of complex traits in livestock species. Our subsequent high-resolution study performed in the target CCR reveals promising candidate mutations to explain some of the identified selection events, including variants in the *RXFP2*, *NPR2*, *NCAPG* and *LCORL* genes, related to the presence of horns and skeletal growth. Further studies are necessary to confirm the possible direct effect of some of the mutations highlighted in this work on the phenotypic variation of traits of interest in sheep.

## Additional files

**Additional file 1.** Phenotypic, production and reproductive traits of Spanish Churra and Australian Merino sheep breeds.

**Additional file 2.** Description of the population structure analyses performed with the 50K-chip genotypes of the samples considered in this study.

**Additional file 3.** **Figure S1.** Graphical representation of the principal component analysis (PCA) performed with Eigensoft for the final of 50K-Chip genotypes analysed in this study for 238 fine wool Merino [Australian Industry Merino (n = 88), Australian Merino (n = 50) and Australian Poll Merino (n = 98)] and 278 Spanish Churra individuals. **Figure S2.** Graphical representation of the results of the cross-validation approach performed with the Admixture software to determine the best K-value. **Figure S3.** Graphical representation of the proportion of membership of each of the analysed populations for K = 2 as obtained with the Admixture_v1.3 software.

**Additional file 4.** Description of the extent of the linkage disequilibrium and block structure based on the analysis of the 50K-chip genotypes of the samples considered in this study.

**Additional file 5: Figure S4.** Average linkage disequilibrium (LD) as a function of genomic distance between markers based on the Churra and Australian Merino 50K-Chip genotypes analyzed in this study. The LD values (y-axis), provided as D’ and r2, are plotted against inter-marker distance bins (x- axis). For each case, the total number of marker pairs were assigned according to their physical distance into 14 categories: < 10 Kb, 10-20 Kb, 20-40 Kb, 40-60 Kb, 60-100 Kb, 200-500 Kb, 0.5-1 Mb, 1-2 Mb, 2-5 Mb, 5-10 Mb, 10-20 Mb, 20-50 Mb or > 50 Mb.

**Additional file 6: Table S2.** List of candidate genes considered in relation to wool-related traits.

**Additional file 7: Table S3.** Whole-genome sequence datasets analyzed in this study.

**Additional file 8: Table S4.** Signatures of selection identified by the genetic differentiation analysis performed between the Australian Merino (Australian Industry Merino, Australian Merino and Australian Poll Merino) and the Churra populations analysed in this study.

**Additional file 9: Table S5.** Selection signals identified by the analysis of observed heterozygosity performed in the Australian Merino populations analysed in the present study.

**Additional file 10: Table S6.** Selection signals identified by the analysis of observed heterozygosity performed in the Churra population analysed in the present study.

**Additional file 11: Table S7.** Selection signals identified by the analysis performed with the hapFLK software (P-value < 0.001) for the Australian Merino and Churra samples analysed in the present study.

**Additional file 12: Table S8.** Signatures of selection identified by the cross-population extended haplotype homozygosity (XP-EHH) analysis performed between the Merino (Australian Industry Merino, Australian Merino and Australian Poll Merino) and the Churra populations analysed in this study (P-value < 0.001).

**Additional file 13: Table S9.** List of genes extracted from the five convergence candidate regions (CCR) identified in this study as positive selection genomic regions in the fine wool Australian Merino lines.

**Additional file 14: Table S10.** List of genes extracted from the five convergence candidate regions (CCR) identified in this study as positive selection genomic regions in the Spanish Churra breed.

**Additional file 15: Table S11.** List of positional candidate genes included in the identified convergence candidate regions (CCR) that were highlighted by our survey with a list of 1459 genes including candidate genes related to traits of interest in sheep.

**Additional file 16: Table S12.** Correspondence of the convergence candidate regions (CCR) identified in our study with previously published genetic effects (QTL and associations) with phenotypic traits of interest in sheep according to the AnimalQTLdb (http://www.animalgenome.org/cgi-bin/QTLdb/OA/index).

**Additional file 17: Table S13.** Results of the four selection sweep mapping analyses between Spanish Merino and Spanish Churra sheep as a validation procedure. The selection signals identified by genetic differentiation (*F*_ST_), reduction of heterozygosity (ObsHtz), and haplotype-based methods (hapFLK andn XP-EHH) were labeled following the same criteria as for the core analyses between Australian Merino and Spanish Churra breeds.

**Additional file 18.** **Figure S5.** Graphical representation of the genetic differentiation analysis (a), and the analysis of reduced heterozygosity (b, c) when analysing the validation dataset “Spanish Merino [34] vs Spanish Churra”. **Figure S6.** Graphical representation of the selection sweep mapping analyses performed with the two haplotype-based methods used in this work, performed with the hapFLK (a) and the rehh (XP-EHH analysis) (b) software, for the validation dataset considered in the present work (Spanish Merino [34] vs Spanish Churra sheep breeds).

**Additional file 19: Table S14.** Convergence candidate regions (CCR) of selection sweeps identified in the validation survey by contrasting genotypes of the Spanish Merino and Spanish Churra sheep breeds. The CCR were defined based on the overlapping between significant signatures of selection (SS) identified by the different individual analysis methods implemented in this study.

**Additional file 20: Table S15.** Allele frequencies for the genetic variants (SNPs and indels) identified from the analysis of 28 WGSeq datasets (15 Churras and 13 Australian Merino) within the 18 genomic regions identified as CCR in the present study.

**Additional file 21: Table S16.** Characterization of the intragenic SNP variations identified, through the processing of the WGSeq datasets considered, within each of the 18 convergence candidate regions (CCR) identified according to the annotation performed with the eVEP software (ensembl Variant Effect Predictor; for further information about the column field names see http://www.ensembl.org/info/docs/tools/vep/vep_formats.html).

**Additional file 22: Table S17.** Summary of the samples included in the “PRJEB14685” Project (high-quality variant calls from the Sheep genomes project - Run1) of the EVA repository (http://www.ebi.ac.uk/ena/data/view/PRJEB14685) carrying the Australian Merino-related allele for the three missense mutations identified in the present study within the candidate convergence region CCR12 (OAR6: 37164263-38580198 bp).

## References

[1] Maijala K, Piper L, Ruvinsky A (1997). Genetic aspects of domestication, common breeds and their origin. The genetics of sheep.

[2] Larson G, Fuller DQ (2014). The evolution of animal domestication. Annu Rev Ecol Evol Syst.

[3] Clutton-Brock J (1981). Domesticated animals from early times.

[4] Fraser AF (1985). Evolution of domesticated animals.

[5] Smith JM, Haigh J (2007). The hitch-hiking effect of a favourable gene. Genet Res.

[6] Kaplan NL, Hudson RR, Langley CH (1989). The “hitchhiking effect” revisited. Genetics.

[7] Wiener P, Wilkinson S (2011). Deciphering the genetic basis of animal domestication. Proc Biol Sci.

[8] Weir BS, Cockerham CC (1984). Estimating F-statistics for the analysis of population structure. Evolution.

[9] Rubin CJ, Zody MC, Eriksson J, Meadows JRS, Sherwood E, Webster MT (2010). Whole-genome resequencing reveals loci under selection during chicken domestication. Nature.

[10] Wiener P, Pong-Wong R (2011). A regression-based approach to selection mapping. J Hered.

[11] Fu YX (1997). Statistical tests of neutrality of mutations against population growth, hitchhiking and background selection. Genetics.

[12] Galtier N, Depaulis F, Barton NH (2000). Detecting bottlenecks and selective sweeps from DNA sequence polymorphism. Genetics.

[13] Hudson RR, Kreitman M, Aguadé M (1987). A test of neutral molecular evolution based on nucleotide data. Genetics.

[14] Nielsen R, Hellmann I, Hubisz M, Bustamante C, Clark AG (2007). Recent and ongoing selection in the human genome. Nat Rev Genet.

[15] Pritchard JK, Pickrell JK, Coop G (2010). The genetics of human adaptation: hard sweeps, soft sweeps, and polygenic adaptation. Curr Biol.

[16] Wilson BA, Petrov DA, Messer PW (2014). Soft selective sweeps in complex demographic scenarios. Genetics.

[17] Gutierrez-Gil B, Arranz JJ, Wiener P (2015). An interpretive review of selective sweep studies in Bos taurus cattle populations: identification of unique and shared selection signals across breeds. Front Genet.

[18] Wilkinson S, Lu ZH, Megens HJ, Archibald AL, Haley C, Jackson IJ (2013). Signatures of diversifying selection in European pig breeds. PLoS Genet.

[19] Gutierrez-Gil B, Arranz JJ, Pong-Wong R, Garcia-Gamez E, Kijas J, Wiener P (2014). Application of selection mapping to identify genomic regions associated with dairy production in sheep. PLoS One.

[20] Moon S, Kim TH, Lee KT, Kwak W, Lee T, Lee SW (2015). A genome-wide scan for signatures of directional selection in domesticated pigs. BMC Genomics.

[21] Kijas JW, Lenstra JA, Hayes B, Boitard S, Porto Neto LR, San Cristobal M (2012). Genome-wide analysis of the World’s sheep breeds reveals high levels of historic mixture and strong recent selection. PLoS Biol.

[22] Fariello MI, Servin B, Tosser-Klopp G, Rupp R, Moreno C, International Sheep Genomics Consortium (2014). Selection signatures in worldwide sheep populations. PLoS One.

[23] Fariello MI, Boitard S, Naya H, SanCristobal M, Servin B (2013). Detecting signatures of selection through haplotype differentiation among hierarchically structured populations. Genetics.

[24] Kijas JW (2014). Haplotype-based analysis of selective sweeps in sheep. Genome.

[25] Moradi MH, Nejati-Javaremi A, Moradi-Shahrbabak M, Dodds KG, McEwan JC (2012). Genomic scan of selective sweeps in thin and fat tail sheep breeds for identifying of candidate regions associated with fat deposition. BMC Genet.

[26] McRae KM, McEwan JC, Dodds KG, Gemmell NJ (2014). Signatures of selection in sheep bred for resistance or susceptibility to gastrointestinal nematodes. BMC Genomics.

[27] Liu Z, Ji Z, Wang G, Chao T, Hou L, Wang J (2016). Genome-wide analysis reveals signatures of selection for important traits in domestic sheep from different ecoregions. BMC Genomics.

[28] Lv FH, Agha S, Kantanen J, Colli L, Stucki S, Kijas JW (2014). Adaptations to climate-mediated selective pressures in sheep. Mol Biol Evol.

[29] Kim E-S, Elbeltagy AR, Aboul-Naga AM, Rischkowsky B, Sayre B, Mwacharo JM (2016). Multiple genomic signatures of selection in goats and sheep indigenous to a hot arid environment. Heredity (Edinb).

[30] Manunza A, Cardoso TF, Noce A, Martínez A, Pons A, Bermejo LA (2016). Population structure of eleven Spanish ovine breeds and detection of selective sweeps with BayeScan and hapFLK. Sci Rep.

[31] Ciani E, Crepaldi P, Nicoloso L, Lasagna E, Sarti FM, Moioli B (2014). Genome-wide analysis of Italian sheep diversity reveals a strong geographic pattern and cryptic relationships between breeds. Anim Genet.

[32] Beynon SE, Slavov GT, Farré M, Sunduimijid B, Waddams K, Davies B (2015). Population structure and history of the Welsh sheep breeds determined by whole genome genotyping. BMC Genet.

[33] Diez-Tascon C, Littlejohn RP, Almeida PAR, Crawford AM (2000). Genetic variation within the Merino sheep breed: analysis of closely related populations using microsatellites. Anim Genet.

[34] Ciani E, Lasagna E, D’Andrea M, Alloggio I, Marroni F, Ceccobelli S (2015). Merino and Merino-derived sheep breeds: a genome-wide intercontinental study. Genet Sel Evol.

[35] Lewis W, Balderstone S, Bowman J (2006). Events that shaped Australia.

[36] Fogarty NM, Safari E, Taylor PJ, Murray W (2003). Genetic parameters for meat quality and carcass traits and their correlation with wool traits in Australian Merino sheep. Aust J Agric Res.

[37] Gardner GE, Williams A, Siddell J, Ball AJ, Mortimer S, Jacob RH (2010). Using Australian sheep breeding values to increase lean meat yield percentage. Anim Prod Sci.

[38] Raadsma HW, Gray GD, Woolaston RR (1998). Breeding for disease resistance in Merino sheep in Australia. Rev Sci Tech.

[39] de la Fuente LF, Fernández G, San Primitivo F (1995). Breeding programme for the Spanish Churra sheep breed. Cahier Options Méditerranéennes.

[40] Miguélez E, Zumalacárregui JM, Osorio MT, Figueira AC, Fonseca B, Mateo J (2008). Quality traits of suckling-lamb meat covered by the protected geographical indication “Lechazo de Castilla y León” European quality label. Small Ruminant Res..

[41] Sambrook J, Russell DW. Purification of nucleic acids by extraction with phenol:chloroform. CSH Protoc. 2006;2006:pii: pdb.prot4455. 10.1101/pdb.prot4455 22485786

[42] Purcell S, Neale B, Todd-Brown K, Thomas L, Ferreira MAR, Bender D (2007). PLINK: a tool set for whole-genome association and population-based linkage analyses. Am J Hum Genet.

[43] Sheep genome assembly v3.1. Available from: http://www.ensembl.org/Ovis_aries/Info/Index.

[44] Patterson N, Price AL, Reich D (2006). Population structure and Eigenanalysis. PLoS Genet.

[45] Alexander DH, Novembre J, Lange K (2009). Fast model-based estimation of ancestry in unrelated individuals. Genome Res.

[46] Akey JM, Zhang G, Zhang K, Jin L, Shriver MD (2002). Interrogating a high-density SNP map for signatures of natural selection. Genome Res.

[47] Akey JM, Ruhe AL, Akey DT, Wong AK, Connelly CF, Madeoy J (2010). Tracking footprints of artificial selection in the dog genome. Proc Natl Acad Sci USA.

[48] Vaysse A, Ratnakumar A, Derrien T, Axelsson E, Rosengren Pielberg G, Sigurdsson S (2011). Identification of genomic regions associated with phenotypic variation between dog breeds using selection mapping. PLoS Genet.

[49] Rubin CJ, Megens HJ, Martinez Barrio A, Maqbool K, Sayyab S, Schwochow D (2012). Strong signatures of selection in the domestic pig genome. Proc Natl Acad Sci USA.

[50] Stainton JJ, Haley CS, Charlesworth B, Kranis A, Watson K, Wiener P (2015). Detecting signatures of selection in nine distinct lines of broiler chickens. Anim Genet.

[51] Bonhomme M, Chevalet C, Servin B, Boitard S, Abdallah J, Blott S (2010). Detecting selection in population trees: the Lewontin and Krakauer test extended. Genetics.

[52] Gautier M, Vitalis R (2012). rehh: an R package to detect footprints of selection in genome-wide SNP data from haplotype structure. Bioinformatics.

[53] Sabeti PC, Varilly P, Fry B, Lohmueller J, Hostetter E, Cotsapas C (2007). Genome-wide detection and characterization of positive selection in human populations. Nature.

[54] Barrett JC, Fry B, Maller J, Daly MJ (2005). Haploview: analysis and visualization of LD and haplotype maps. Bioinformatics.

[55] Tang K, Thornton KR, Stoneking M (2007). A new approach for using genome scans to detect recent positive selection in the human genome. PLoS Biol.

[56] Hu ZL, Park CA, Wu XL, Reecy JM (2013). Animal QTLdb: an improved database tool for livestock animal QTL/association data dissemination in the post-genome era. Nucleic Acids Res.

[57] Kinsella RJ, Kahari A, Haider S, Zamora J, Proctor G, Spudich G (2011). Ensembl BioMarts: a hub for data retrieval across taxonomic space. Database (Oxford).

[58] Stenn KS, Paus R (2001). Controls of hair follicle cycling. Physiol Rev.

[59] Wang Z, Zhang H, Yang H, Wang S, Rong E, Pei W (2014). Genome-wide association study for wool production traits in a Chinese Merino sheep population. PLoS One.

[60] Liu N, Li H, Liu K, Yu J, Cheng M, De W (2014). Differential expression of genes and proteins associated with wool follicle cycling. Mol Biol Rep.

[61] SRA Toolkit documentation. Available from: http://www.ncbi.nlm.nih.gov/Traces/sra/?view=software.

[62] Kijas J, Brauning R, Clarke SM, McCulloch A, Cockett NE, Saunders G (2016). Launching SheepGenomesDB: 100 million variants from nearly 500 sheep genomes. J Anim Sci.

[63] McKenna A, Hanna M, Banks E, Sivachenko A, Cibulskis K, Kernytsky A (2010). The Genome Analysis Toolkit: a MapReduce framework for analyzing next-generation DNA sequencing data. Genome Res.

[64] Li H, Handsaker B, Wysoker A, Fennell T, Ruan J, Homer N (2009). The sequence alignment/map format and SAMtools. Bioinformatics.

[65] Andrews S. FastQC: a quality control tool for high throughput sequence data. 2010. Available from: http://www.bioinformatics.babraham.ac.uk/projects/fastqc.

[66] Bolger AM, Lohse M, Usadel B (2014). Trimmomatic: a flexible trimmer for Illumina sequence data. Bioinformatics.

[67] Li H, Durbin R (2009). Fast and accurate short read alignment with Burrows–Wheeler transform. Bioinformatics.

[68] Li H (2011). A statistical framework for SNP calling, mutation discovery, association mapping and population genetical parameter estimation from sequencing data. Bioinformatics.

[69] Institute Broad. Picard tool, version 1.128. Available from: http://broadinstitute.github.io/picard/.

[70] DePristo MA, Banks E, Poplin R, Garimella KV, Maguire JR, Hartl C (2011). A framework for variation discovery and genotyping using next-generation DNA sequencing data. Nat Genet.

[71] Cingolani P, Patel VM, Coon M, Nguyen T, Land SJ, Ruden DM (2012). Using *Drosophila melanogaster* as a model for genotoxic chemical mutational studies with a new program, SnpSift. Front Genet.

[72] Narasimhan V, Danecek P, Scally A, Xue Y, Tyler-Smith C, Durbin R (2016). BCFtools/RoH: a hidden Markov model approach for detecting autozygosity from next-generation sequencing data. Bioinformatics.

[73] Danecek P, Auton A, Abecasis G, Albers CA, Banks E, DePristo MA (2011). The variant call format and VCFtools. Bioinformatics.

[74] McLaren W, Pritchard B, Rios D, Chen Y, Flicek P, Cunningham F (2010). Deriving the consequences of genomic variants with the Ensembl API and SNP Effect Predictor. Bioinformatics.

[75] Kumar P, Henikoff S, Ng PC (2009). Predicting the effects of coding non-synonymous variants on protein function using the SIFT algorithm. Nat Protoc.

[76] Sheep reference genome Oar_4.0. Available from: https://www.ncbi.nlm.nih.gov/genome?term=ovisaries.

[77] Bai Y, Sartor M, Cavalcoli J (2012). Current status and future perspectives for sequencing livestock genomes. J Anim Sci Biotechnol.

[78] Boitard S, Boussaha M, Capitan A, Rocha D, Servin B (2016). Uncovering adaptation from sequence data: Lessons from genome resequencing of four cattle breeds. Genetics.

[79] Kardos M, Luikart G, Bunch R, Dewey S, Edwards W, McWilliam S (2015). Whole-genome resequencing uncovers molecular signatures of natural and sexual selection in wild bighorn sheep. Mol Ecol.

[80] Herrero-Medrano JM, Megens HJ, Groenen MAM, Bosse M, Pérez-Enciso M, Crooijmans RPMA (2014). Whole-genome sequence analysis reveals differences in population management and selection of European low-input pig breeds. BMC Genomics.

[81] Kijas JW, Porto-Neto L, Dominik S, Reverter A, Bunch R, McCulloch R (2014). Linkage disequilibrium over short physical distances measured in sheep using a high-density SNP chip. Anim Genet.

[82] Chitneedi PK, Arranz JJ, Suárez-Vega A, García-Gámez E, Gutiérrez-Gil B (2017). Estimations of linkage disequilibrium, effective population size and ROH-based inbreeding coefficients in Spanish Churra sheep using imputed high-density SNP genotypes. Anim Genet.

[83] Meadows JRS, Chan EKF, Kijas JW (2008). Linkage disequilibrium compared between five populations of domestic sheep. BMC Genet.

[84] Al-Mamun HA, Clark SA, Kwan P, Gondro C (2015). Genome-wide linkage disequilibrium and genetic diversity in five populations of Australian domestic sheep. Genet Sel Evol.

[85] Sánchez Belda A, Sánchez Trujillano MC. Razas ovinas españolas. Publicaciones de Extensión Agraria, Ministerio de Agricultura, Pesca y Alimentación; 1986.

[86] Arranz JJ, Bayón Y, San Primitivo F (1998). Genetic relationships among Spanish sheep using microsatellites. Anim Genet.

[87] García-Gámez E, Sahana G, Gutiérrez-Gil B, Arranz J-J (2012). Linkage disequilibrium and inbreeding estimation in Spanish Churra sheep. BMC Genet.

[88] Sabeti PC, Schaffner SF, Fry B, Lohmueller J, Varilly P, Shamovsky O (2006). Positive natural selection in the human lineage. Science.

[89] González-Rodríguez A, Munilla S, Mouresan EF, Cañas-Álvarez JJ, Díaz C, Piedrafita J (2016). On the performance of tests for the detection of signatures of selection: a case study with the Spanish autochthonous beef cattle populations. Genet Sel Evol.

[90] Gholami M, Reimer C, Erbe M, Preisinger R, Weigend A, Weigend S (2015). Genome scan for selection in structured layer chicken populations exploiting linkage disequilibrium information. PLoS One.

[91] Sims D, Sudbery I, Ilott NE, Heger A, Ponting CP (2014). Sequencing depth and coverage: key considerations in genomic analyses. Nat Rev Genet.

[92] Matika O, Riggio V, Anselme-Moizan M, Law AS, Pong-Wong R, Archibald AL (2016). Genome-wide association reveals QTL for growth, bone and in vivo carcass traits as assessed by computed tomography in Scottish Blackface lambs. Genet Sel Evol.

[93] Raadsma HW, Thomson PC, Zenger KR, Cavanagh C, Lam MK, Jonas E (2009). Mapping quantitative trait loci (QTL) in sheep. I. A new male framework linkage map and QTL for growth rate and body weight. Genet Sel Evol.

[94] Cavanagh CR, Jonas E, Hobbs M, Thomson PC, Tammen I, Raadsma HW (2010). Mapping quantitative trait loci (QTL) in sheep. III. QTL for carcass composition traits derived from CT scans and aligned with a meta-assembly for sheep and cattle carcass QTL. Genet Sel Evol.

[95] Al-Mamun HA, Kwan P, Clark SA, Ferdosi MH, Tellam R, Gondro C (2015). Genome-wide association study of body weight in Australian Merino sheep reveals an orthologous region on OAR6 to human and bovine genomic regions affecting height and weight. Genet Sel Evol.

[96] Lango Allen H, Estrada K, Lettre G, Berndt SI, Weedon MN, Rivadeneira F (2010). Hundreds of variants clustered in genomic loci and biological pathways affect human height. Nature.

[97] Tetens J, Widmann P, Kühn C, Thaller G (2013). A genome-wide association study indicates LCORL/NCAPG as a candidate locus for withers height in German Warmblood horses. Anim Genet.

[98] Eberlein A, Takasuga A, Setoguchi K, Pfuhl R, Flisikowski K, Fries R (2009). Dissection of genetic factors modulating fetal growth in cattle indicates a substantial role of the *non*-*SMC condensin I complex, subunit G* (*NCAPG*) gene. Genetics.

[99] Sahana G, Höglund JK, Guldbrandtsen B, Lund MS (2015). Loci associated with adult stature also affect calf birth survival in cattle. BMC Genet.

[100] Setoguchi K, Watanabe T, Weikard R, Albrecht E, Kühn C, Kinoshita A (2011). The SNP c.1326T > G in the *non*-*SMC condensin I complex, subunit G* (*NCAPG*) gene encoding a p.Ile442Met variant is associated with an increase in body frame size at puberty in cattle. Anim Genet.

[101] Setoguchi K, Furuta M, Hirano T, Nagao T, Watanabe T, Sugimoto Y (2009). Cross-breed comparisons identified a critical 591-kb region for bovine carcass weight QTL (CW-2) on chromosome 6 and the Ile-442-Met substitution in *NCAPG* as a positional candidate. BMC Genet.

[102] Lindholm-Perry AK, Sexten AK, Kuehn LA, Smith TPL, King DA, Shackelford SD (2011). Association, effects and validation of polymorphisms within the *NCAPG*-*LCORL* locus located on BTA6 with feed intake, gain, meat and carcass traits in beef cattle. BMC Genet.

[103] Vaysse A, Ratnakumar A, Derrien T, Axelsson E, Rosengren Pielberg G, Sigurdsson S (2011). Identification of genomic regions associated with phenotypic variation between dog breeds using selection mapping. PLoS Genet.

[104] Abo-Ismail MK, Vander Voort G, Squires JJ, Swanson KC, Mandell IB, Liao X (2014). Single nucleotide polymorphisms for feed efficiency and performance in crossbred beef cattle. BMC Genet.

[105] Gowen LC, Petersen DN, Mansolf AL, Qi H, Stock JL, Tkalcevic GT (2003). Targeted disruption of the *osteoblast/osteocyte factor 45* gene (*OF45*) results in increased bone formation and bone mass. J Biol Chem.

[106] Wei J, Geale PF, Sheehy PA, Williamson P (2012). The impact of ABCG2 on bovine mammary epithelial cell proliferation. Anim Biotechnol.

[107] Sheehy PA, Riley LG, Raadsma HW, Williamson P, Wynn PC (2009). A functional genomics approach to evaluate candidate genes located in a QTL interval for milk production traits on BTA6. Anim Genet.

[108] Kühn C, Weikard R, Widmann P. Metabolomics: a pathway for improved understanding of genetic modulation of mammalian growth and tissue deposition. In: Proceedings of the 10th world congress on genetics applied to livestock production: 17–22 August 2014; Vancouver. 2014.

[109] Liu Y, Duan X, Chen S, He H, Liu X, Liu Y (2015). *NCAPG* is differentially expressed during longissimus muscle development and is associated with growth traits in Chinese Qinchuan beef cattle. Genet Mol Biol.

[110] UniProt Consortium (2008). The Universal Protein Resource (UniProt). Nucleic Acids Res.

[111] Martínez-Cerezo S, Sañudo C, Panea B, Medel I, Delfa R, Sierra I (2005). Breed, slaughter weight and ageing time effects on physico-chemical characteristics of lamb meat. Meat Sci.

[112] Campo MM, Olleta J, Sañudo C. Características de la carne de cordero con especial atención al Ternasco de Aragón. Agencia Aragonesa de Seguridad Alimentaria. 2008.

[113] Allain D, Miari S, Usai MG, Barillet F, Sechi T, Sechi S, et al. SNP mapping of QTL affecting wool traits in a sheep backcross Sarda-Lacaune resource population. In: Proceedings of the 64th annual meeting of the European Federation of Animal Science: 26–30 August 2013; Nantes. 2013.

[114] Cano M, Allain D, Foulquié D, Moreno C, Mulsant P, François D, et al. Fine mapping of birthcoat type in the Romane breed sheep. In: Proceedings of the 64th Annual Meeting of the European Federation of Animal Science: 26–30 August 2013; Nantes. 2013.

[115] Olivier W, Olivier J, Greyling A. Quantifying the relationship between birth coat score and wool traits in Merino sheep. In: Proceedings of the 10th world conference on animal production: 23–28 November 2008; Cape Town. 2008.

[116] Liu F, Visser M, Duffy DL, Hysi PG, Jacobs LC, Lao O (2015). Genetics of skin color variation in Europeans: genome-wide association studies with functional follow-up. Hum Genet.

[117] Johnston SE, McEwan J, Pickering NK, Kijas JW, Beraldi D, Pilkington JG (2011). Genome-wide association mapping identifies the genetic basis of discrete and quantitative variation in sexual weaponry in a wild sheep population. Mol Ecol.

[118] Allais-Bonnet A, Grohs C, Medugorac I, Krebs S, Djari A, Graf A (2013). Novel insights into the bovine polled phenotype and horn ontogenesis in Bovidae. PLoS One.

[119] Wiedemar N, Tetens J, Jagannathan V, Menoud A, Neuenschwander S, Bruggmann R (2014). Independent polled mutations leading to complex gene expression differences in cattle. PLoS One.

[120] Yuan FP, Li X, Lin J, Schwabe C, Bullesbach EE, Rao CV (2010). The role of RXFP2 in mediating androgen-induced inguinoscrotal testis descent in LH receptor knockout mice. Reproduction.

[121] Scott DJ, Rosengren KJ, Bathgate RAD (2012). The different ligand-binding modes of relaxin family peptide receptors RXFP1 and RXFP2. Mol Endocrinol.

[122] Wiedemar N, Drögemüller C (2015). A, 1.8-kb insertion in the 3′-UTR of RXFP2 is associated with polledness in sheep. Anim Genet.

[123] Lühken G, Krebs S, Rothammer S, Küpper J, Mioč B, Russ I (2016). The 1.78-kb insertion in the 3′-untranslated region of *RXFP2* does not segregate with horn status in sheep breeds with variable horn status. Genet Sel Evol.

[124] Bartels CF, Bükülmez H, Padayatti P, Rhee DK, van Ravenswaaij-Arts C, Pauli RM (2004). Mutations in the *transmembrane natriuretic peptide receptor NPR*-*B* impair skeletal growth and cause acromesomelic dysplasia, type Maroteaux. Am J Hum Genet.

[125] Vasques GA, Amano N, Docko AJ, Funari MFA, Quedas EPS, Nishi MY (2013). Heterozygous mutations in *natriuretic peptide receptor*-*B* (*NPR2*) gene as a cause of short stature in patients initially classified as idiopathic short stature. J Clin Endocrinol Metab.

[126] García-Gámez E, Gutiérrez-Gil B, Sahana G, Sánchez JP, Bayón Y, Arranz JJ (2012). GWA analysis for milk production traits in dairy sheep and genetic support for a QTN influencing milk protein percentage in the LALBA gene. PLoS One.

[127] Saatchi M, Schnabel RD, Taylor JF, Garrick DJ (2014). Large-effect pleiotropic or closely linked QTL segregate within and across ten US cattle breeds. BMC Genomics.

