# Supplementary material for: High-resolution analysis of selection sweeps identified between fine-wool Merino and coarse-wool Churra sheep breeds
Source: Genet Sel Evol. 2017 Nov 7;49:81. doi: 10.1186/s12711-017-0354-x (PMC5674817; doi:10.1186/s12711-017-0354-x)
Supplement: Supplementary file 1 — Additional file 1. Phenotypic, production and reproductive traits of Spanish Churra and Australian Merino sheep breeds. [file 12711_2017_354_MOESM1_ESM.docx]

**Additional file 1 for “High resolution analysis of selection sweeps identified between fine-wool Merino and coarse-wool Churra sheep breeds”**

**Authors:** Beatriz Gutiérrez-Gil, Cristina Esteban-Blanco, Pamela Wiener, Praveen Krishna Chitneedi, Aroa Suarez-Vega, Juan-José Arranz

**Additional file 1: Table S1.** Phenotypic, production and reproductive traits of Spanish Churra and Australian Merino sheep breeds.

| **Traits** | **Spanish Churra** | **Australian Merino** |  |
| --- | --- | --- | --- |
| Geographic Origin | South West Europe | South West Europe |  |
| Weight | Males: 75-85 Kg ^1^  Females: 60-65 Kg ^1^ | Males: 80-90 Kg ^2^  Females: 57 Kg ^3^ |  |
| Stature | Females: 67,6 cm ^4^  (height to rump tallest point) | Males: 71,0 cm ^5^  Females: 61,0 cm ^5^ |  |
| Weight at birth | 3.92 Kg ^6^ | 3.63 Kg ^7^; 4.1 Kg ^8^ |  |
| Tail shape | Thin tail | Thin tail |  |
| Horn shape | Rams can be polled or horned  (can carry knowb or scurs).  Most of ewes are pooled | Rams can be polled or horned  *AustralianPollMerino: derived from Merinos where hornlessness was selected for. |  |
|  |  | |  |
| Wool type | Coarse  30-40 microns ^4^ | Extremely fine  16.1-17.5 microns (ultra-fine)  17.6-18.5 microns (super-fine)  18.6-19.5 microns (fine) |  |
| Coat pigmentation | White with black spots around the eyes, and on the ears, nose, belly and distal end of the legs | White face and white fleece. Lack of pigmentation |  |
| Prolificacy  (lambs born/ewe lambing) | 1,35 ^4^ | 1,06 ^9^ |  |
| Production and Purpose | Double purpose breed  (milk and suckling lamb production) | Wool with selection pressure to reduce susceptibility to parasites ^10^ |  |

^1^ Fuentes-García F.C., Sanchez-Sánchez J.M, Gonzalo-Abascal C. 2006. Tratado de Etnología Animal (Razas de rumiantes y monogástricos). Diego Marín. Librero Editor.ISBN-13: 978-84-8425-492-8.

^2^ http://www.vet.unicen.edu.ar/ActividadesCurriculares/Zootecnia/images/apunte-ovinos-e28093-base-animal-razas.pdf

^3^ Rose G1, Kause A, Mulder HA, van der Werf JH, Thompson AN, Ferguson MB, van Arendonk JA. 2013. Merino ewes can be bred for body weight change to be more tolerant to uncertain feed supply. J Anim Sci. 91(6):2555-65.

^4^ Fernando de la Fuente, Personal communication. Breeding programme coordinator of the National Association of Churra Breeders (ANCHE).

^5^<https://docs.google.com/document/d/1o7n_CK8h3UNhUdJwmButE7ATN-6i5K2K5fLmIcr9Dks/edit>

^6^ Gutierrez J., De la Fuente L.F., San Primitivo F., Rodríguez R. 2006. Factors affecting growth and carcass quality of Churra lambs. XXXI Jornadas Científicas y X internacionales Sociedad Española de Ovinotecnia y Caprinotecnia (SEOC), pp 79-82. Zamora, 20-22 September 2006.

^7^ Hatcher S., Atkins K. D. and Safari E. 2009. Phenotypic aspects of lamb survival in Australian Merino sheep. J Anim Sci. 87(9):2781-2790.

^8^ Fogarty N, Hopkins D, H. 1998. Lambs production from diverse genotypes. Final report 1994-1997. Cowra Agricultural Research and Advisory Station. NSW Agriculture.

^9^ Bianchi O.G., Garibotto C.G., Lamarca B.M. 2014. Estudio comparativo de hembras finnish landrace x merino australiano vs. merino australiano: desempeño reproductivo. AbanicoVet, 4 (1): 33-37. Available at <http://new.medigraphic.com/cgi-bin/resumen.cgi?IDARTICULO=49037>

^10^ Kijas J.W., Lenstra J.A., Hayes B., Boitard S., Porto Neto L.R., San Cristobal M., Servin B., McCulloch R., Whan V., Gietzen K., Paiva S., Barendse W., Ciani E., Raadsma H., McEwan J., Dalrymple B. 2012. International Sheep Genomics Consortium Members. Genome-wide analysis of the world's sheep breeds reveals high levels of historic mixture and strong recent selection. PLoS Biol. 10(2):e1001258.
