# Supplementary material for: High-resolution analysis of selection sweeps identified between fine-wool Merino and coarse-wool Churra sheep breeds
Source: Genet Sel Evol. 2017 Nov 7;49:81. doi: 10.1186/s12711-017-0354-x (PMC5674817; doi:10.1186/s12711-017-0354-x)
Supplement: Supplementary file 2 — Additional file 2. Description of the population structure analyses performed with the 50K-chip genotypes of the samples considered in this study. [file 12711_2017_354_MOESM2_ESM.docx]

**Additional file 2 for “High resolution analysis of selection sweeps identified between fine-wool Merino and coarse-wool Churra sheep breeds”**

**Authors:** Beatriz Gutiérrez-Gil, Cristina Esteban-Blanco, Pamela Wiener, Praveen Krishna Chitneedi, Aroa Suarez-Vega, Juan-José Arranz

**Additional file 2.** Description of the population structure analyses performed with the 50K-Chip genotypes of the samples considered in the present study.

1. **MATERIAL AND METHODS**
   1. **Population structure analysis methodology**

First, a Principal Component Analysis (PCA) of allele sharing performed using *smartpca* implemented in Eigensoft [44] was performed for the 50K-Chip genotypes of the three Merino fine wool breeds [Australian Industry Merino (n = 88), Australian Merino (n = 50) and Australian Poll Merino (n = 98)] and Spanish Churra sheep (n = 278) considered in this study. From this analysis, the proportion of variance explained by each component was obtained by dividing the eigenvalue corresponding to each component by the sum of all eigenvalues identified (for a total of 20 PC estimated).

In addition, we performed an analysis with Admixture_v1.3, which calculates maximum likelihood estimates of individuals ancestries based on the data provided by multiple loci [45]. The analysis was performed for K values ranging from 1 to 10.

1. **RESULTS**

**2.1. Population structure analysis results**

The results of the PCA of allele sharing performed with Eigensoft were plotted to show direct comparison of Principal Component 1 (PC1) against PC2 to PC5 (Additional file 3: Figure S1). The two largest principal components (A) clearly separated Spanish Churra sheep from the three Merino breeds. Interestingly, the three Merino breeds were more homogenous than Churra sheep. The graphical representation of the other

Based on this analysis, PC1 explained 25.44% of the genotypic variance, PC2 explained 7.61% and PC3, PC4 and PC5 explained 5.55%, 5.25% and 4.98% of the variance respectively.

For the different K-values tested in the Admixture analysis the profiles of the likelihood and the cross-validation errors did not show a clear peak for any given value of K. Hence, we identified the more accurate value of K by estimating DeltaK (L’’), which is defined as L''(K) = abs(L'(K)-L'(K+1). Hence, based on DeltaK estimation the value of K =2 was chose as the best estimated number of population (Additional file 3: Figure S2). This criterion was supported by the graphical representation of the individual ancestries shown in Additional file 3: Figure S3.
