# Supplementary material for: High-resolution analysis of selection sweeps identified between fine-wool Merino and coarse-wool Churra sheep breeds
Source: Genet Sel Evol. 2017 Nov 7;49:81. doi: 10.1186/s12711-017-0354-x (PMC5674817; doi:10.1186/s12711-017-0354-x)

**Additional file 3 for “High resolution analysis of selection sweeps identified between fine-wool Merino and coarse-wool Churra sheep breeds”**

**Authors:** Beatriz Gutiérrez-Gil, Cristina Esteban-Blanco, Pamela Wiener, Praveen Krishna Chitneedi, Aroa Suarez-Vega, Juan-José Arranz

**Figure S1.** Graphical representation of the Principal Component Analysis (PCA) performed with Eigensoft for the final of 50K-Chip genotypes analysed in this study for a total of 238 fine wool Merino [Australian Industry Merino (n = 88), Australian Merino (n = 50) and Australian Poll Merino (n = 98)] and 278 Spanish Churra individuals.


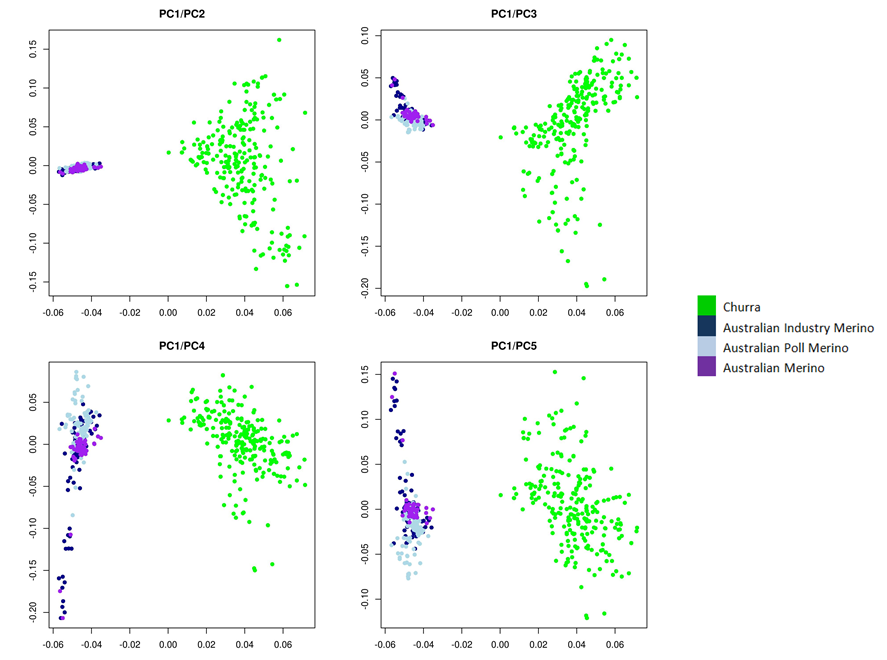


**Figure S2.** Graphical representation of the results of the cross-validation approach performed with the Admixture software to determine the best K-value.


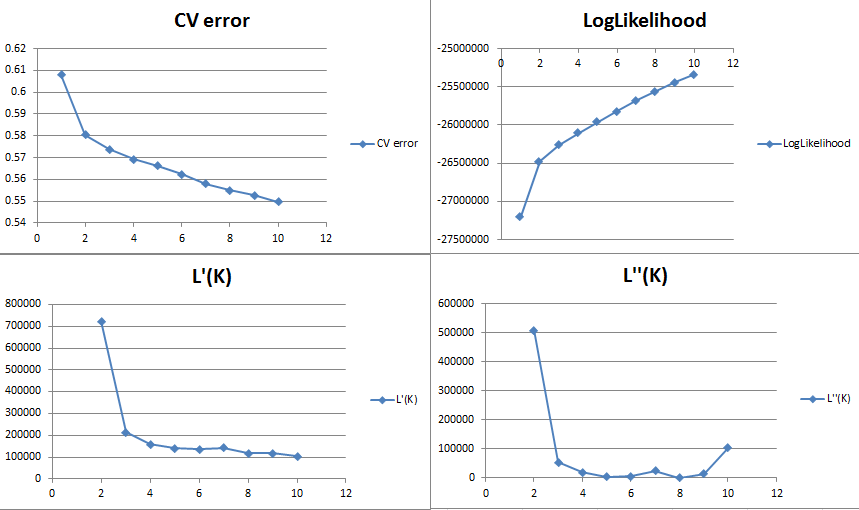


**Figure S3.** Graphical representation of the proportion of membership of each of the analysed populations for K = 2 as obtained with the Admixture_v1.3 software.


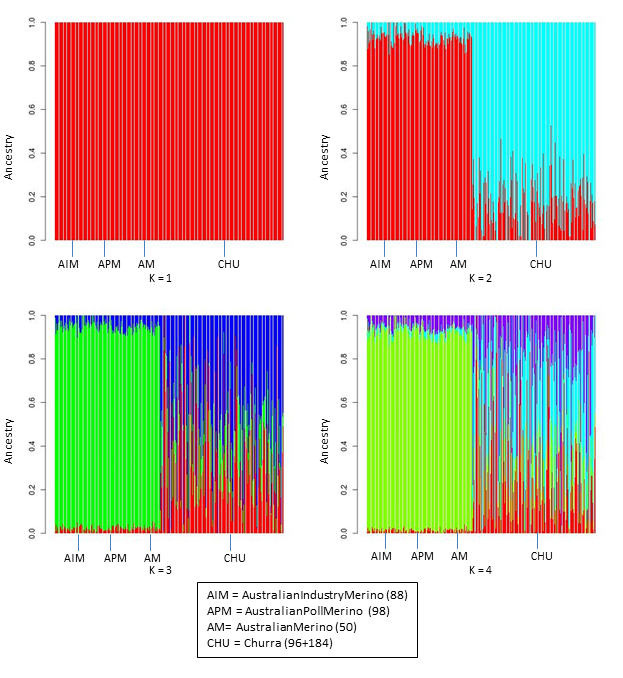

Supplement: Supplementary file 3 — Additional file 3. Figure S1. Graphical representation of the principal component analysis (PCA) performed with Eigensoft for the final of 50K-Chip genotypes analysed in this study for 238 fine wool Merino [Australian Industry Merino (n = 88), Australian Merino (n = 50) and Australian Poll Merino (n = 98)] and 278 Spanish Churra individuals. Figure S2. Graphical representation of the results of the cross-validation approach performed with the Admixture software to determine the best K-value. Figure S3. Graphical representation of the proportion of membership of each of the analysed populations for K = 2 as obtained with the Admixture_v1.3 software. [file 12711_2017_354_MOESM3_ESM.docx]
