# Supplementary material for: High-resolution analysis of selection sweeps identified between fine-wool Merino and coarse-wool Churra sheep breeds
Source: Genet Sel Evol. 2017 Nov 7;49:81. doi: 10.1186/s12711-017-0354-x (PMC5674817; doi:10.1186/s12711-017-0354-x)
Supplement: Supplementary file 4 — Additional file 4. Description of the extent of the linkage disequilibrium and block structure based on the analysis of the 50K-chip genotypes of the samples considered in this study. [file 12711_2017_354_MOESM4_ESM.docx]

**Additional file 4 for “High resolution analysis of selection sweeps identified between fine-wool Merino and coarse-wool Churra sheep breeds”**

**Authors:** Beatriz Gutiérrez-Gil, Cristina Esteban-Blanco, Pamela Wiener, Praveen Krishna Chitneedi, Aroa Suarez-Vega, Juan-José Arranz

**Additional file 4.** Description of the extent of the linkage disequilibrium and block structure based on the analysis of the 50K-Chip genotypes of the samples considered in the present study.

1. **MATERIAL AND METHODS**
   1. **Linkage disequilibrium (LD) estimations and study of haplotype block structure in the Churra and Merino populations under study.**

After the QC of the 50K-Chip genotypes, the Haploview_v4.2 software [54], was used to studied the LD extent and haplotype block structure for the Churra and Merino populations considered here following the approaches previously used by García-Gámez et al. [87]. For each marker pair LD measurements were estimated and expressed as *r^2^* and *D’*. Using *r^2^* and *D’* estimates, we analyzed the extent and decay of LD across the genome, by calculating the half length of *r^2^*, which represents the LD decays to half of its maximum length. LD was also estimated over shorter distances i.e. from 1 Kb to 300 Kb by averaging *r^2^* and *D’* in 1-Kb bins.

Haploview_v4.2 was also used to define the haplotype blocks present in the genome. The method followed for block definition was Gabriel's block definition according to which a pair of SNPs is defined to be in ‘strong LD’ if the one-sided upper 95% confidence bound of *D’* is higher than 0.98 and if the lower bound is above 0.7. In contrast, ‘strong evidence for historical recombination’ is defined if the upper confidence bound on *D’* is less than 0.9.

1. **RESULTS**

**2.1. Extent of LD and haplotype block structure of the Churra and Merino population under study.**

Taking into account all the possible marker pairs within each chromosome we estimated the LD parameters in the Churra and Merino populations considered in the present study. Based on the mean *r^2^* and *D’* values estimated for the different marker distance bins (Additional File 4. Table a), the two breeds showed a similar pattern of decrease LD with increased physical distance between SNPs, with similar average *r^2^* values for markers up to 40 Kb apart (Average *r^2^* values at 10Kb: 0.280 and 0.274 (*D’* = 0.779 and 0.765) for Churra and Merino, respectively; Average *r^2^* values at 20-40 Kb: 0.148-0.141 (*D’* = 0.612 and 0.579) for Churra and Merino, respectively), although for longer distances the Merino breed showed lower LD values than Churra (For 40-60 Kb, average *r^2^* values were 0.114 (*D’* = 0.553) and 0.102 (*D’* = 0.508) in Churra and Merino, respectively (See Additional file 5: Figure S4). The half-length decay value of LD (*r^2^*) was 0.1398 in Churra and 0.137 in Merino (Additional File 4. Table a), which corresponds to distances between SNPs of 40-60 Kb (roughly 50 Kb). The LD pattern results reported here agree with previous reports showing that both Australian Merino and Churra are two of the breeds showing one of the shortest extents of LD among domestic sheep breeds [81, 82].

A summary of the distribution, size, number and SNPs involved in the haploblocks detected per chromosome are presented in Additional File 4. Table b. In Churra, a total of 1,963 haploblocks spanning 46,17 Mb (1,88%) of the autosomal genome were detected. In Merino, a total of 1,939 haploblocks spanning 40,46 Kb (1,65%) of the autosomal genome were detected. The average block size was 23.521 Kb in Churra and 20.865 Kb in Merino. In Churra, the range of the block length was from 0.036 Kb (OAR14, 2 SNPs) to 1033.56 Kb (OAR10, 10 SNPs), whereas in Merino the range was from 0.88 Kb (OAR22, 2 SNPs) to 779.239 kb (OAR10, 12 SNPs). The proportion of blocks of size 50 Kb or longer was 43.65% and 50.59% in Merino and Churra respectively.

**Additional File 4. Table a.** Mean linkage disequilibrium (LD) estimated among synthetic SNPs at different physical distances based on the 50K-Chip genotypes of the Churra and Merino populations analyzed in this study. The average LD estimates are provided in *r^2^* and *D'* units for each population.

|  | Churra | | Merino | |
| --- | --- | --- | --- | --- |
| Bin | Mean *D’* | Mean_*r’* | Mean *D’* | Mean_*r’* |
| 10Kb | 0.779087 | 0.279606 | 0.765246 | 0.27491 |
| 10-20Kb | 0.704018 | 0.209623 | 0.678581 | 0.202974 |
| 20-40Kb | 0.611633 | 0.148405 | 0.57897 | 0.141119 |
| 40-60Kb | 0.553151 | 0.114045 | 0.507669 | 0.102736 |
| 60-100Kb | 0.502251 | 0.0875057 | 0.447854 | 0.0749253 |
| 100-200Kb | 0.44993 | 0.0632393 | 0.381944 | 0.0485553 |
| 200-500Kb | 0.415743 | 0.0497272 | 0.332132 | 0.032713 |
| 500Kb-1Mb | 0.396553 | 0.0439929 | 0.293478 | 0.0244605 |
| 1-2Mb | 0.376665 | 0.0386931 | 0.256824 | 0.0180311 |
| 2-5Mb | 0.340734 | 0.0301397 | 0.213489 | 0.011712 |
| 5-10Mb | 0.297736 | 0.0214038 | 0.185587 | 0.00825397 |
| 10-20Mb | 0.258397 | 0.0147856 | 0.171823 | 0.00681194 |
| 20-50Mb | 0.221637 | 0.00978934 | 0.162525 | 0.00600837 |
| 50Mb | 0.207459 | 0.00818352 | 0.159112 | 0 |

**Additional File 4. Table b.** Summary of the block structure found in the two sheep populations studied in the present study.

|  |  | Churra | | | Merino | | |
| --- | --- | --- | --- | --- | --- | --- | --- |
| Chr. |  | Number of blocks | Max. block length | Min block length | Number of blocks | Max. block length | Min block length |
| 1 |  | 251 | 222.153 | 2.385 | 236 | 214.514 | 2.692 |
| 2 |  | 240 | 652.585 | 4.024 | 247 | 652.585 | 3.657 |
| 3 |  | 182 | 453.421 | 4.223 | 181 | 246.354 | 4.047 |
| 4 |  | 105 | 226.932 | 2.064 | 104 | 231.233 | 2.064 |
| 5 |  | 83 | 151.073 | 3.751 | 94 | 181.712 | 3.734 |
| 6 |  | 101 | 228.131 | 3.724 | 94 | 258.673 | 2.024 |
| 7 |  | 94 | 157.193 | 4.055 | 96 | 322.007 | 2.198 |
| 8 |  | 78 | 182.981 | 2.615 | 73 | 111.131 | 2.615 |
| 9 |  | 94 | 488.533 | 3.658 | 90 | 392.388 | 3.658 |
| 10 |  | 72 | 1033.56 | 2.462 | 75 | 779.239 | 2.654 |
| 11 |  | 39 | 201.071 | 5.761 | 37 | 174.969 | 5.761 |
| 12 |  | 67 | 257.178 | 1.945 | 64 | 93.082 | 1.945 |
| 13 |  | 59 | 161.957 | 3.746 | 49 | 188.435 | 3.746 |
| 14 |  | 36 | 118.51 | 0.036 | 36 | 122.023 | 1.07 |
| 15 |  | 55 | 83.767 | 2.131 | 62 | 107.413 | 2.131 |
| 16 |  | 50 | 134.485 | 2.462 | 46 | 178.245 | 2.462 |
| 17 |  | 47 | 186.29 | 4.769 | 45 | 122.776 | 4.769 |
| 18 |  | 46 | 170.919 | 5.434 | 47 | 129.016 | 5.079 |
| 19 |  | 52 | 161.357 | 5.732 | 49 | 29.57 | 5.732 |
| 20 |  | 35 | 145.708 | 3.209 | 31 | 185.134 | 6.328 |
| 21 |  | 24 | 19.238 | 2.843 | 24 | 21.39 | 2.843 |
| 22 |  | 47 | 229.598 | 0.88 | 48 | 171.448 | 0.88 |
| 23 |  | 32 | 326.097 | 6.712 | 27 | 62.236 | 6.152 |
| 24 |  | 19 | 19.096 | 4.383 | 25 | 19.096 | 4.383 |
| 25 |  | 35 | 205.277 | 6.02 | 39 | 270.545 | 4.199 |
| 26 |  | 20 | 17.93 | 0.922 | 20 | 63.669 | 0.922 |
