# Supplementary material for: High-resolution analysis of selection sweeps identified between fine-wool Merino and coarse-wool Churra sheep breeds
Source: Genet Sel Evol. 2017 Nov 7;49:81. doi: 10.1186/s12711-017-0354-x (PMC5674817; doi:10.1186/s12711-017-0354-x)

**Additional file 5 for “High resolution analysis of selection sweeps identified between fine-wool Merino and coarse-wool Churra sheep breeds”**

**Authors:** Beatriz Gutiérrez-Gil, Cristina Esteban-Blanco, Pamela Wiener, Praveen Krishna Chitneedi, Aroa Suarez-Vega, Juan-José Arranz

**Figure S4.** Average linkage disequilibrium (LD) as a function of genomic distance between markers based on the Churra and Australian Merino 50K-Chip genotypes analyzed in the present study. The LD values (y-axis), provided as *D’* and *r^2^*, are plotted against inter-marker distance bins (x- axis). For each case, the total number of marker pairs were assigned according to their physical distance into 14 categories: < 10 Kb, 10-20 Kb, 20-40 Kb, 40-60 Kb, 60-100 Kb, 200-500 Kb, 0.5-1 Mb, 1-2 Mb, 2-5 Mb, 5-10 Mb, 10-20 Mb, 20-50 Mb or > 50 Mb.


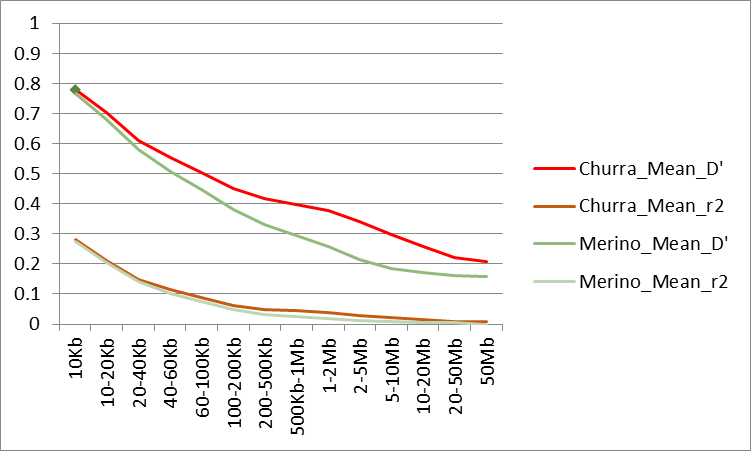

Supplement: Supplementary file 5 — Additional file 5: Figure S4. Average linkage disequilibrium (LD) as a function of genomic distance between markers based on the Churra and Australian Merino 50K-Chip genotypes analyzed in this study. The LD values (y-axis), provided as D’ and r2, are plotted against inter-marker distance bins (x- axis). For each case, the total number of marker pairs were assigned according to their physical distance into 14 categories: < 10 Kb, 10-20 Kb, 20-40 Kb, 40-60 Kb, 60-100 Kb, 200-500 Kb, 0.5-1 Mb, 1-2 Mb, 2-5 Mb, 5-10 Mb, 10-20 Mb, 20-50 Mb or > 50 Mb. [file 12711_2017_354_MOESM5_ESM.docx]
