# Supplementary material for: High-resolution analysis of selection sweeps identified between fine-wool Merino and coarse-wool Churra sheep breeds
Source: Genet Sel Evol. 2017 Nov 7;49:81. doi: 10.1186/s12711-017-0354-x (PMC5674817; doi:10.1186/s12711-017-0354-x)
Supplement: Supplementary file 18 — Additional file 18. Figure S5. Graphical representation of the genetic differentiation analysis (a), and the analysis of reduced heterozygosity (b, c) when analysing the validation dataset “Spanish Merino [34] vs Spanish Churra”. Figure S6. Graphical representation of the selection sweep mapping analyses performed with the two haplotype-based methods used in this work, performed with the hapFLK (a) and the rehh (XP-EHH analysis) (b) software, for the validation dataset considered in the present work (Spanish Merino [34] vs Spanish Churra sheep breeds). [file 12711_2017_354_MOESM18_ESM.docx]

**Additional file 18 for “High resolution analysis of selection sweeps identified between fine-wool Merino and coarse-wool Churra sheep breeds”**

**Authors:** Beatriz Gutiérrez-Gil, Cristina Esteban-Blanco, Pamela Wiener, Praveen Krishna Chitneedi, Aroa Suarez-Vega, Juan-José Arranz

**Figure S5. Graphical representation of the genetic differentiation analysis (a), and the analysis of reduced heterozygosity (b, c) when analyzing the validation dataset “Spanish Merino *vs* Spanish Churra” considered in the present study.**

a) F_ST_ values obtained across the whole genome (averaged in sliding windows of 9 SNPs) when contrasting the 50K-Chip pooled genotypes of the Spanish Merino samples provided by Ciani et al. [34] and a subset of Spanish Churra samples considered in this work. The horizontal line indicates the top 0.5^th^ percent threshold of the F_ST_-distribution; b) and c) Genome-wide distribution of observed heterozygosity values (averaged over a sliding window of 9 SNPs) for the pooled genotypes of the Spanish Merino (b) and the Spanish Churra (c) samples considered as a validation dataset in the present work. The horizontal lines indicate the bottom 0.5^th^ percent thresholds of the heterozygosity distributions.


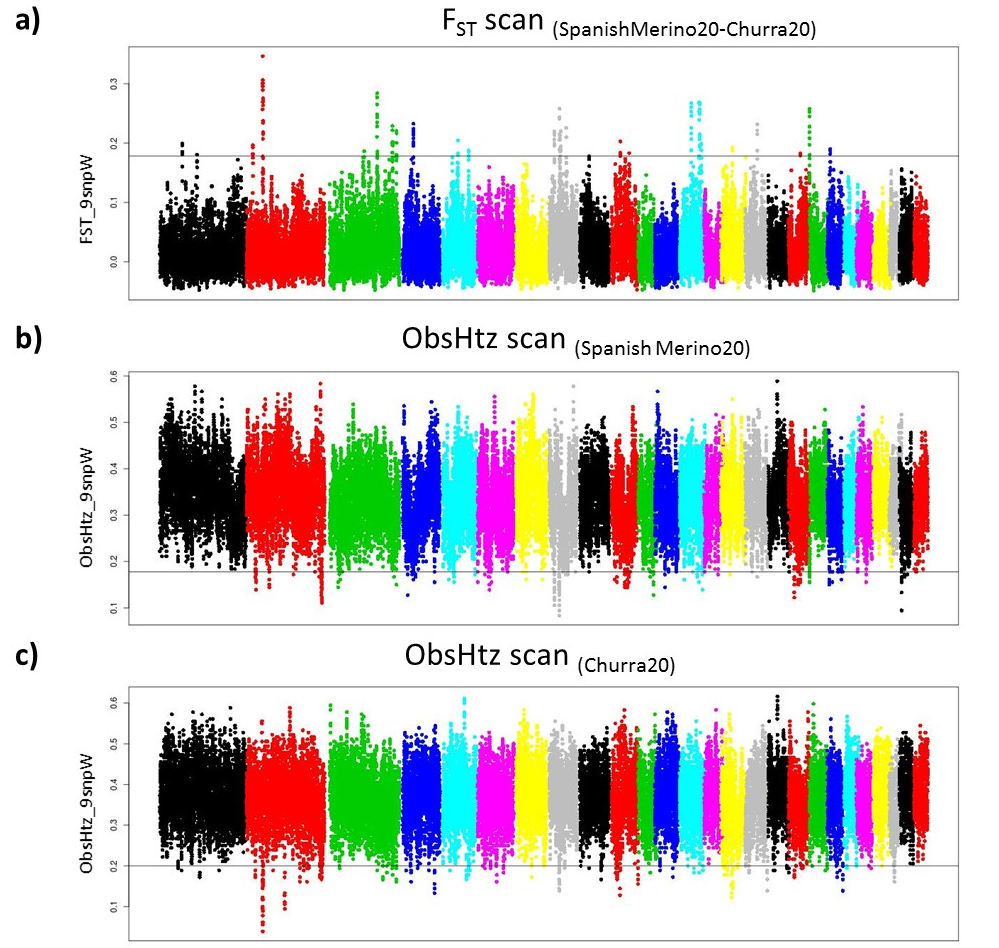


**Figure S6. Graphical representation of the selection sweep mapping analyses performed with the two haplotype-based methods used in this work, performed with the hapFLK (a) and the *rehh* (XP-EHH analysis) (b) software, for the validation dataset considered in the present work (Spanish Merino *vs* Spanish Churra sheep breeds).** Genome-wide distribution of the log (1/P-value) obtained from each analysis are represented in the Y-axis. The horizontal lines represent the significance threshold considered (P < 0.001).

**
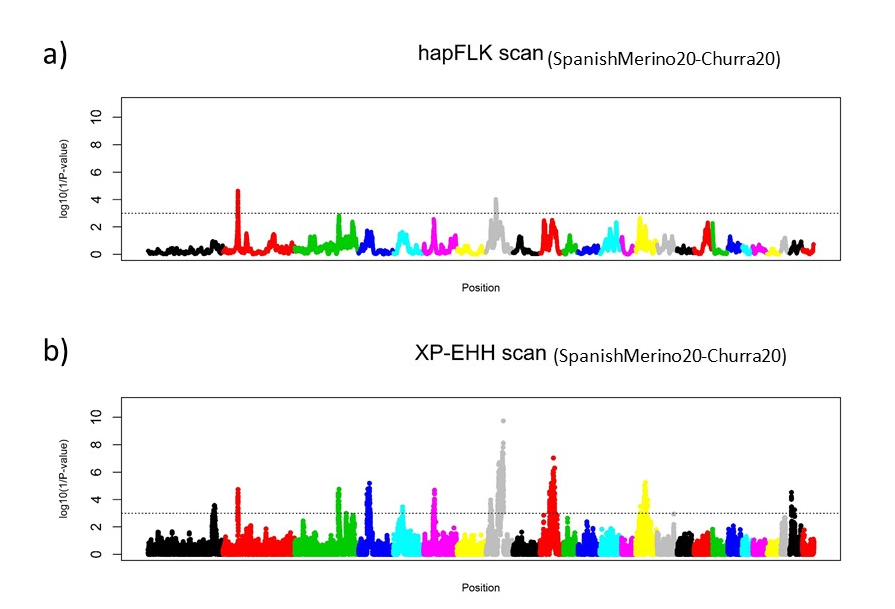
**
